# Mechanisms of Cell Entry by dsRNA Viruses: Insights for Efficient Delivery of dsRNA and Tools for Improved RNAi-Based Pest Control

**DOI:** 10.3389/fphys.2021.749387

**Published:** 2021-11-11

**Authors:** Luc Swevers, Dimitrios Kontogiannatos, Anna Kolliopoulou, Feifei Ren, Min Feng, Jingchen Sun

**Affiliations:** ^1^Insect Molecular Genetics and Biotechnology, Institute of Biosciences and Applications, National Centre for Scientific Research “Demokritos”, Athens, Greece; ^2^Guangdong Provincial Key Laboratory of Agro-Animal Genomics and Molecular Breeding, College of Animal Science, South China Agricultural University, Guangzhou, China

**Keywords:** dsRNA virus, entry mechanism, arbo-reovirus, plant reovirus, *Cypovirus*, RNAi, pest control, viral-like particle

## Abstract

While RNAi is often heralded as a promising new strategy for insect pest control, a major obstacle that still remains is the efficient delivery of dsRNA molecules within the cells of the targeted insects. However, it seems overlooked that dsRNA viruses already have developed efficient strategies for transport of dsRNA molecules across tissue barriers and cellular membranes. Besides protecting their dsRNA genomes in a protective shell, dsRNA viruses also display outer capsid layers that incorporate sophisticated mechanisms to disrupt the plasma membrane layer and to translocate core particles (with linear dsRNA genome fragments) within the cytoplasm. Because of the perceived efficiency of the translocation mechanism, it is well worth analyzing in detail the molecular processes that are used to achieve this feat. In this review, the mechanism of cell entry by dsRNA viruses belonging to the *Reoviridae* family is discussed in detail. Because of the large amount of progress in mammalian versus insect models, the mechanism of infections of reoviruses in mammals (orthoreoviruses, rotaviruses, orbiviruses) will be treated as a point of reference against which infections of reoviruses in insects (orbiviruses in midges, plant viruses in hemipterans, insect-specific cypoviruses in lepidopterans) will be compared. The goal of this discussion is to uncover the basic principles by which dsRNA viruses cross tissue barriers and translocate their cargo to the cellular cytoplasm; such knowledge subsequently can be incorporated into the design of dsRNA virus-based viral-like particles for optimal delivery of RNAi triggers in targeted insect pests.

## Introduction

RNA interference (RNAi) gradually has matured as a promising and safe strategy for insect pest management that can be applied successfully to control susceptible (typically coleopteran) species (Christiaens et al., 2020; Fletcher et al., 2020). RNAi insecticides became reality when Monsanto Co (now part of Bayer’s crop science division) received approval of the U.S. Environmental Protection Agency (EPA) for DvSnf7 dsRNA as a plant protection trait against western corn rootworm (*Diabrotica virgifera*) in transgenic maize (U.S. EPA, 2017; Head et al., 2017). In SmartStax Pro maize, dsRNA targeting the *snf7* gene of *Diabrotica* together with two *Bacillus thuringiensis* Cry proteins are expressed as transgenes, a strategy also designated as host-induced gene silencing (HIGS; Ghag, 2017). However, the use of transgenic crops as a source for RNAi insecticides has considerable limitations, including long development time, difficulties of genetic transformation for many crops and high resistance of the public opinion in several markets, most notably the European Union, that have led to the adoption of protracted regulatory procedures (EFSA GMO Panel, 2011, 2018).

Currently, non-transformative approaches of RNAi, known as spray-induced gene silencing (SIGS; San Miguel and Scott, 2016; Wytinck et al., 2020) are gaining momentum, especially since the cost of production of dsRNA has dropped to levels that make the strategy economically feasible (Cagliari et al., 2019; Shaffer, 2020; Rodrigues et al., 2021). Topically applied dsRNA products may also be close to commercialization against susceptible pests such as the Colorado potato beetle (Cagliari et al., 2019; Rodrigues et al., 2021).

However, a huge barrier for application of dsRNA as a sprayed insecticide or plant protection trait remains the efficient uptake by the targeted pests (Kontogiannatos et al., 2021; Silver et al., 2021). As a natural product, dsRNA is sensitive to environmental degradation and enzymatic breakdown within the insect (Singh et al., 2017; Bachman et al., 2020). Furthermore, to penetrate the plasma or endosomal membrane is a major challenge for negatively charged hydrophilic molecules such as dsRNA that requires considerable engineering efforts (Ni et al., 2019). In some coleopteran insects, most notably *D. virgifera* and *Leptinotarsa decemlineata* mentioned above, gene silencing and related toxic effects can be achieved by low concentrations of exogenous dsRNA, a process which is not very well understood (Ivashuta et al., 2015) but could be related to efficient endosomal escape (Shukla et al., 2016; Vélez and Fishilevich, 2018). However, for the majority of insect pests, environmental RNAi is not efficient and requires modifications and encapsulations to boost efficiency (Fletcher et al., 2020; Kontogiannatos et al., 2021). From this point of view, the strategy of SIGS seems to be at an advantage since dsRNAs can be easily manipulated during spray formulation (SIGS approach) while such procedures are more challenging to achieve *in planta* (HIGS approach).

Different non-transformative technologies for delivery of dsRNA were tested against various insect pests that include nanoparticles based on liposomes, polymers, dendrimers and cell-penetrating peptides (Whyard et al., 2009; Gillet et al., 2017; Christiaens et al., 2018; Yan et al., 2021). While safety features of dsRNAs are mostly determined by the degree of cross-hybridization with homologous mRNAs in non-target organisms (Romeis and Widmer, 2020), the potential toxicity and persistence of the carrier moieties (nanoparticles) may need to be tested independently (Sajid et al., 2015). A key safety feature may be the use of natural and biodegradable materials, for instance chitosan (Zhang et al., 2010c) and sheet-like clay nanoparticles (“BioClay”; Mitter et al., 2017). Engineered bacteria and viruses that produce dsRNAs (Palli, 2014; Kolliopoulou et al., 2017; Taning et al., 2018) have the potential to be used in spray applications but face regulatory barriers, since they are considered as genetically modified organisms. The permeability, stability and potency of small interfering RNAs (siRNAs) can be improved by chemical modifications (an approach that is used in human medicine; Dowdy, 2017) but are associated with high production costs and potential side effects (Garber, 2017). Finally, little is known about the uptake mechanism of dsRNA formulations by midgut tissue in insect larvae after feeding (Kunte et al., 2020). Endocytosis has been implicated as the major mechanism for uptake of naked dsRNA from the culture medium in cell lines and from the body cavity in tissues following hemolymph injection (Saleh et al., 2006; Xiao et al., 2015). However, more research is required how the complexation of dsRNA with carrier molecules or its encapsulation in nanoparticles will affect the pathways of cellular entry in the insect midgut after oral uptake.

## Diversity of dsRNA Viruses

It seems overlooked that nature already has invented efficient vehicles to transport dsRNA molecules to the interior of cells: dsRNA viruses that have evolved to protect and deliver linear dsRNA genome fragments into host cells during infection. DsRNA viruses comprise a polyphyletic group that originated from positive sense ssRNA viruses on multiple occasions (Koonin et al., 2015). Well-studied dsRNA viruses that infect insects belong to the families of *Reoviridae* and *Birnaviridae*, which have a totally different origin.

Several genera of reoviruses that infect insects include arthropod-borne viruses (arboviruses), *i.e.*, viruses that use biting insects such as mosquitoes or ticks as vectors and may cause disease in humans or livestock (Kuno and Chang, 2005), or plant viruses that are transmitted by leafhoppers or planthoppers and are pathogenic to plants, including many crops (Nault, 1997; Hogenhout et al., 2008). In each case, viral replication occurs in both the insect vectors and the mammalian or plant hosts.

Arboviral reoviruses of the genus *Orbivirus*, mainly Bluetongue virus (BTV) that is transmitted by *Culicoides* midges and causes disease in ruminants (Samy and Peterson, 2016), have received a lot of research interest, although mostly focused in their mammalian hosts (Patel and Roy, 2014). Arbo-reoviruses belonging to the genera *Coltivirus* and *Seadornavirus* (Dolja and Koonin, 2018) are less well studied.

Plant reoviruses include the genera *Fijivirus*, *Oryzavirus* and *Phytoreovirus* and their transmission by hemipteran vectors has been studied extensively (Wei and Li, 2016). On the other hand, reoviruses of the genera *Cypovirus* (formerly known as cytoplasmic polyhedrosis viruses or CPV) (Mertens et al., 1999), *Dinovernavirus* (Attoui et al., 2005), and *Idnoreovirus* (Graham et al., 2008) only infect insects.

Birnaviruses are classified within the alphavirus-like superfamily which reflects its evolution from this group of positive sense ssRNA viruses (Koonin et al., 2015). While virions of reoviruses generally have a double (or triple) capsid structure (with cypoviruses as notable exception) and regularly encapsidate 10 to 12 linear dsRNA genomic fragments (Miyazaki et al., 2008), birnaviruses contain 2 dsRNA genome segments within a single-shelled particle (Delmas et al., 2019). Viruses of the genus *Entomobirnavirus* infect flies (*Drosophila*) and mosquitoes (Tesh et al., 2020).

Recently, toti-like (‘Toti-Chryso clade’ of dsRNA viruses) and partiti-like viruses (‘Partiti-Picobirna clade’) were identified in insects by meta-transcriptome analyses (Shi et al., 2016; Dolja and Koonin, 2018). Both toti- and partiti-like viruses are small dsRNA viruses with a persistent lifestyle that are highly promiscuous and can spread to a high variety of different hosts, including fungi, insects and unicellular parasites. While most toti-like viruses typically encode only an RNA-dependent RNA polymerase and a capsid protein on monopartite dsRNA genomes, partiti-like viruses that infect arthropods can diverge from the canonical bipartite dsRNA genome organization and contain different numbers of dsRNA genome segments (Dolja and Koonin, 2018). Interestingly, partiti-like viruses can play differential ecological roles in closely related insect species, from mutualistic-symbiotic to parasitic (Xu et al., 2020). Because of their simple organization, the capsid proteins of toti-like and partiti-like viruses form single-shelled particles but their structures are different than those from other dsRNA viruses (Naitow et al., 2002; Pan et al., 2009).

## DsRNA Viruses as Efficient Carriers and Delivery Vehicles of dsRNA Molecules

All dsRNA viruses mentioned above are non-enveloped and therefore may be less vulnerable to environmental conditions. In the absence of a lipid envelope, reoviruses have evolved strategies for cell entry that are based on the properties and structures of their capsid proteins (Zhang et al., 2010b, 2016; Abdelhakim et al., 2014). When produced in high amounts, reovirus capsid proteins can assemble into genetically inert viral-like particles (VLPs) that may have preserved the capacity for specific interactions with cell surfaces and efficient cell membrane penetration (Kolliopoulou et al., 2017; Zhao et al., 2018). If protocols can be developed for packaging non-reproductive dsRNA, reoviral (or birnaviral) VLPs could be employed as specific and efficient vehicles for delivery of RNAi in animals, including insects.

Because of this prospect, it will be interesting to explore which mechanisms reoviruses use to transverse cellular membranes and deliver cargo in the cellular cytoplasm. Most of this research has been performed with mammalian cell lines and significant progress was obtained in the understanding of the mechanism by which reoviruses of the genera *Orthoreovirus*, *Rotavirus* and *Orbivirus* acquire access to the cytoplasm of mammalian cells (Baker and Prasad, 2010; Danthi et al., 2013; Zhang et al., 2016). Orthoreoviruses and rotaviruses are specific for vertebrates and are transmitted by the respiratory (orthoreoviruses) and fecal-oral routes (both orthoreo- and rotaviruses) (Day, 2009; Crawford et al., 2018). While rotaviruses are mostly known for their infection of the small intestinal mucosa and causing gastroenteritis in humans, mainly children (Crawford et al., 2018), orthoreoviruses can also propagate in multiple epithelia from the intestine, lung and bile duct as well as in the central nervous system and leukocytes (Gauvin et al., 2013). As already mentioned, orbiviruses enter the vertebrate organism after the insect or tick bite together with the saliva but are taken up by the insect vectors after feeding of a blood meal via the midgut (Mills et al., 2017). As arboviruses, orbiviruses are also known to infect mosquito cell lines but information about the mechanism of cell entry remains limited compared with mammalian cells (Tan et al., 2001; Roy and Noad, 2006).

Of the insect-specific reoviruses, cypoviruses (CPVs) have been studied most abundantly (Belloncik and Mori, 1998; Swevers et al., 2020). Cypovirus virions exceptionally have a single shell capsid layer, which is similar to the inner core of other reoviruses. However, cypovirus virions typically become embedded in paracrystalline occlusion bodies that are composed of the protein polyhedrin (Ji et al., 2015). Polyhedra with similar properties are also formed by baculoviruses (or nucleopolyhedroviruses) but the polyhedrin proteins are markedly different and accumulate in the nucleus, in contrast to the cytoplasm for cypoviruses. Polyhedra provide protection for the virions in the environment but are disrupted by the high alkaline pH in the larval lepidopteran midgut (Rohrmann, 1986). Studies have been performed about the uptake of CPV virions in cell lines and in the larval midgut (Swevers et al., 2020), but no detailed mechanism was described about how viral particles penetrate cellular membrane barriers to initiate viral transcription and replication in the cytoplasm. A comparative study therefore may be useful, since insights in the mechanisms of reovirus entry in mammalian cells can provide important cues for understanding infection of insect cells by cypoviruses. Mechanistic understanding also can stimulate engineering to achieve new characteristics and improvements to increase specificity and efficiency.

In this review, the mechanisms of cell entry by orthoviruses, rotaviruses and orbiviruses in mammalian cells are first discussed at the molecular and (sub)cellular level regarding cell binding, trafficking by endosomes and cell/endosomal membrane penetration. Avoidance of apoptosis, stimulation of signaling pathways and cellular tropism are also considered. Next, the more limited information of cell entry by reoviruses in insects (orbiviruses in midges, plant viruses in hemipterans, lepidopteran-specific cypoviruses) will be analyzed. Based on the available information, an outline will be presented about the mechanism by which cypoviruses gain access to the cytoplasm of its main target tissue, the midgut epithelial cells, in lepidopteran insects. Although the review will be focused on reoviruses, for which most of the information is available, the potential of birnaviruses will also be briefly discussed in a separate section. The goal of this discussion is to provide a solid background for the establishment of a biotechnological platform that can be used for the production of dsRNA virus-based VLPs for optimal delivery of RNAi in targeted insect pests. An overview of the molecular mechanisms of cell entry by the dsRNA viruses that are discussed is presented in Table 1.

**TABLE 1:** Overview of the molecular mechanisms of cellular entry by diverse dsRNA viruses.

	**Cell attachment**	**Cell internalization**	**Proteolytic processing/Destabilization**	**Site of membrane penetration**	**Signaling pathways**
Mammalian orthoreovirus (*Orthoreovirus*) (mammalian)	Low affinity: Sialylated glycans High Affinity: JAM-A NgR1	Clathrin-mediated endocytosis Caveolar Endocytosis Macropinocytosis β1 integrins	Endosomes: Cathepsin B and L Intestinal lumen: Trypsin, Chymotrypsin	Early and late endosomes	Apoptosis NFκ-B Src kinase TGFβ

Human/Rhesus rotavirus (*Rotavirus*) (mammalian)	Sialoglycans Polymorphic histoblood group antigens Integrins α2β1, αvβ3 and αxβ2 JAM-A	Clathrin- and caveolin-independent (Dynamin, Cholesterol, HSC70) Clathrin-mediated endocytosis Direct penetration	Endosomes: cathepsins B, L and S Intestinal lumen: Trypsin Calcium loss	Mature endosomes (early penetrating) Late endosomes (late penetrating) Plasma membrane	RhoA/ROCK/Myosin Light chain Cation-dependent mannose-6-phosphate receptor ERK PI3K-AKT

Bluetongue virus (*Orbivirus*) (mammalian)	Sialic acids	Clathrin-mediated endocytosis Macropinocytosis	pH (acidification)-dependent	Early and late endosomes	Apoptosis NFκ-B MAPK/ERK

Bluetongue virus (*Orbivirus*) (insect)		Integrin-dependent Clathrin-mediated endocytosis	Midgut lumen: Proteases	Endosomes	

Rice dwarf virus (*Phytoreovirus*) (insect)		Clathrin-mediated endocytosis	pH (acidification)-dependent	Endosomes	Interaction with actin cytoskeleton by helical tubular structures

Rice black-streaked dwarf virus (*Fijivirus*) (insect)		Vesicle-associated membrane protein 7 Vesicle transport VSNARE protein Growth hormone-inducible transmembrane protein			Interaction with actin cytoskeleton by tubular structures

Cypovirus-1 (Cytoplasmic polyhedrosis virus) (*Cypovirus*) (insect)	Cholesterol Gangliosides	Direct penetration Clathrin-mediated endocytosis β integrins	Midgut lumen: Dissolution of polyhedra by alkaline pH	Plasma membrane Endosomes	Apoptosis De-ubiquitination De-SUMO-ylation Src64B-like kinase RACK1

Infectious bursal disease virus (*Avibirnavirus*) (bird)	Hsp90 Annexin II Integrin α4/β1 Immunoglobulin M	Macropinocytosis (clathrin-independent)	Calcium loss pH (acidification)-dependent	Early endosomes	Apoptosis Src kinase

*For more detailed description, see main text.*

## Multiple-Layered Capsid Structure of Mammalian Reoviruses

**FIGURE 1:**
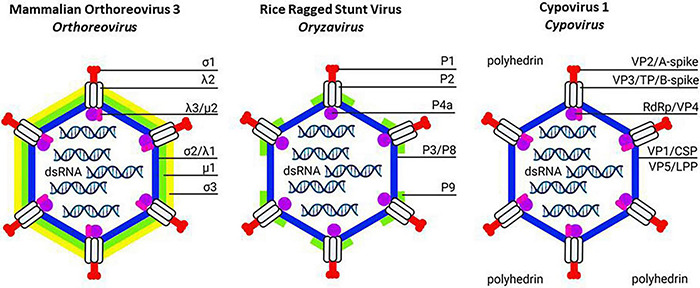
Schematic structure of turreted reoviruses (*Spinareovirinae*): Mammalian orthoreovirus 3 (**Left**; *Orthoreovirus*), Rice ragged stunt virus (**Middle**; *Oryzavirus*) and Cypovirus 1 (**Right**; *Cypovirus*). The transition from double-layered to single-layered particles is illustrated. In orthoreoviruses, the outer capsid consists of the *T* = 13/icosahedral layer of trimers (formed by μ1; green) in complex with the protection protein (σ3; yellow). The *T* = 13/icosahedral layer of trimers is more limited in oryzaviruses (formed by P9; green) and absent in cypoviruses. Instead, cypovirus virions can become embedded in polyhedra formed by a three-dimensional network of polyhedrin protein. Characteristic turrets (formed by λ2, P2 or VP3/TP/B-spike; gray) also serve to anchor the spike proteins at the exterior (formed by σ1, P1 or VP2/A-spike; red) and the transcription complex at the interior (formed by λ3/μ2, P4a or RdRp/VP4; purple-pink). The inner shell with clamp (formed by σ2/λ1, P3/P8 or VP1/CSP-VP5/LPP; blue) encapsulates the dsRNA segments of the genome. Created with BioRender.com.

**FIGURE 2:**
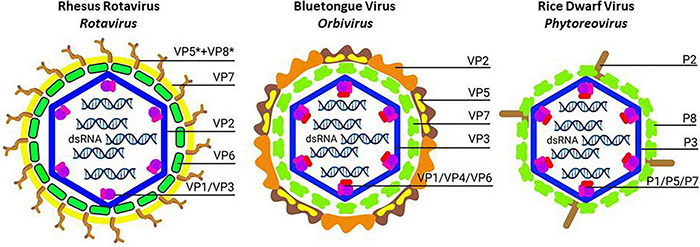
Schematic structure of non-turreted reoviruses (*Sedoreovirinae*): Rhesus rotavirus (**Left**; *Rotavirus*), Bluetongue virus (**Middle**; *Orbivirus*) and Rice dwarf virus (**Right**; *Phytoreovirus*). The transition from triple-layered to double-layered particles is illustrated. In each case, core particles consist of the inner shell (formed by VP2, VP3 or P3; blue) that enclose the dsRNA genome segments together with the *T* = 13/icosahedral layer of trimers (formed by VP6, VP7 or P6; green). The outer capsid layers of Rhesus rotavirus (processed spike protein VP5* + VP8*; brown, and glycoprotein VP7; yellow) and Bluetongue virus (spike protein VP2; light and dark brown, and penetration protein VP5; yellow) provide the mechanism for entry into mammalian cells. For Rice dwarf virus, the spike protein P2 (brown) is involved in membrane destabilization and is necessary for infection of insect vectors after feeding. It is noted that double-layered core particles of Bluetongue virus and Rice dwarf virus are capable to infect insect cells in culture efficiently. For infection of insect cells, trimers in the *T* = 13/icosahedral layer (VP7 or P8; green) function as the penetration proteins. Created with BioRender.com.

Reoviruses are divided in two subfamilies based on the presence of “turrets” (or “spikes”) at the vertices of the capsids: non-turreted *Sedoreovirinae* and turreted *Spinareovirinae* (Andrew et al., 2012). However, both turreted and non-turreted reoviruses share a common thin core capsid shell that creates a large cavity for the packaging of dsRNA genome segments and the transcription machinery (Miyazaki et al., 2008). The reoviral core shell constitutes an ancient feature and is built by 120 copies of thin crescent-shaped proteins that exhibit similar folding among all reoviruses (e.g., λ1 for orthoreoviruses (Reinisch et al., 2000); VP2 for rotavirus (Lawton et al., 1997); VP3 for Bluetongue virus (Grimes et al., 1998; Figures 1, 2 and Tables 2, 3), despite the almost absence of any recognizable homology in the primary protein sequences. When over-expressed, core shell proteinscan assemble spontaneously to icosahedral structures, indicating their importance for the initiation of the assembly process (Labbé et al., 1991; Moss and Nuttall, 1994; Lawton et al., 1997).

**TABLE 2:** Protein and nucleic acid composition of virions of the turreted *Spinareovirinae* subfamily of reoviruses and representative for the genera *Orthoreovirus* (Mammalian orthoreovirus 3: Chandran et al., 2001; Mendeza et al., 2008; Sutton et al., 2020), *Oryzavirus* (Rice ragged stunt virus; Li et al., 1996; Upadhyaya et al., 1996; Miyazaki et al., 2008), and *Cypovirus* (Cypovirus 1: Yu et al., 2011; Li et al., 2017; Zhao et al., 2018).

	**Mammalian**	**Rice ragged**	**Cypovirus 1**
	**orthoreovirus 3**	**stunt virus**	
	**85 nm mature particle**	**70 nm mature particle**	**70 nm mature particle**
**Core Particle**			
**Inner shell**	λ1 (142 kDa, 120 copies)	P3 (101 kDa, 120 copies)	VP1 (CSP) (149 kDa, 120 copies)
**Clamp**	σ2 (47 kDa, 150 copies)	P8 (42 kDa, 120 copies)	VP5 (LPP) (50 kDa, 120 copies)
**Turret (Capping enzyme)**	λ2 (144 kDa, 60 copies)	P2 (B-spike) (133 kDa, 60 copies)	VP3 (TP or B-spike) (120 kDa, 60 copies)
**Outer Layer**			
**Spike**	σ1 (50 kDa, 36 copies)	P1 (A-spike) (138 kDa, ? copies) putative	VP2 (A-spike) (140 kDa, ? copies)
**Layer of trimers (*T* = 13) Penetration**	μ1 (76 kDa, 600 copies)	P9 (39 kDa, 180 copies)	
**Protection protein**	σ3 (41 kDa, 600 copies)		Polyhedrin (28.5 kDa) (forms polyhedra)
**Transcription**			
**RNA-dependent RNA polymerase**	λ3 (142 kDa, 12 copies)	P4a (141 kDa, 12 copies)	RDRP (138.5 kDa, 12 copies)
**Cofactor**	μ2 (83 kDa, 20 copies) NTPase	?	VP4 (63.5 kDa, 12 copies) NTPase
**Genome**	10 dsRNA segments	10 dsRNA segments	10 dsRNA segments

*“Core particles” correspond to transcriptionally competent particles delivered into cytoplasm. Capping enzymes are located in the turrets of viruses belonging to the *Spinavirinae* subfamily. Instead of a layer on the surface of the shell, polyhedrin trimers build a large tridimensional network that form large occlusion bodies (polyhedra) for protection of embedded cypovirus virions.*

**TABLE 3:** Protein and nucleic acid composition of virions of the non-turreted *Sedoreovirinae* subfamily and representative for the genera *Rotavirus* (Rhesus rotavirus: Pesavento et al., 2006; Guglielmi et al., 2010; Arias et al., 2015), *Orbivirus* (Bluetongue virus; Mertens and Diprose, 2004; Mohl and Roy, 2014; Roy, 2017), and *Phytoreovirus* (Rice dwarf virus: Lu et al., 1998; Hagiwara et al., 2003; Nakagawa et al., 2003).

	**Rhesus rotavirus**	**Bluetongue virus**	**Rice dwarf virus**
	**100 nm mature particle**	**90 nm mature particle**	**70 nm mature particle**
**Core Particle**			
**Inner shell**	VP2 (102 kDa, 120 copies)	VP3 (103 kDa, 120 copies)	P3 (114 kDa, 120 copies)
**Layer of trimers (*T* = 13)**	VP6 (45 kDa, 780 copies)	VP7 (39 kDa, 780 copies)	P8 (46 kDa, 780 copies) Penetration
**Outer Layer**			
**Spike**	VP8* (28 kDa, 180 copies)	VP2 (111 kDa, 180 copies)	P2 (127 kDa, 10–30 copies)
**Penetration**	VP5* (60 kDa, 180 copies)	VP5 (59 kDa, 360 copies)	
**Protection protein**	VP7 (34 kDa, 780 copies)		
**Transcription**			
**RNA-dependent RNA polymerase**	VP1 (125 kDa, 12 copies)	VP1 (150 kDa, 12 copies)	P1 (164 kDa, 12 copies)
**Capping enzyme**	VP3 (88 kDa, 12 copies)	VP4 (76 kDa, 24 copies)	P5 (91 kDa, ? copies)
**Cofactor**		VP6 (36 kDa, 72 copies) helicase	P7 (55 kDa, ? copies) nucleic acid-binding
**Genome**	11 dsRNA segments	10 dsRNA segments	12 dsRNA segments

*“Core particles” correspond to transcriptionally competent particles delivered into cytoplasm. In Rhesus rotavirus, the spike protein VP4 becomes proteolytically processed to VP8^∗^ and VP5^∗^ that remain attached to the virion and have roles in cell attachment and membrane penetration, respectively.*

Turreted and non-turreted reoviruses form different phylogenetic groups based on sequence homologies of the RdRp, the only protein for which significant identity/similarity can be found across all reoviruses (Miyazaki et al., 2008). In addition, as indicated by their nomenclature, both groups differ in the structural organization of the surrounding capsid layers.

Mammalian turreted orthoreoviruses have a two-layered capsid. Their core particles are characterized by the assembly of five-fold symmetric turrets that house the mRNA capping enzymes (based on the λ2 turret protein in mammalian orthoreoviruses; Figure 1 and Table 2) and the presence of clamp proteins, that stabilize the capsid shells (σ2 in orthoreovirus; Reinisch et al., 2000). Thus, in mammalian turreted orthoviruses, the core particle that becomes transcriptionally active after delivery in the cytoplasm of the host cells consists of the capsid proteins λ1 (inner shell), λ2 (turret) and σ2 (clamp) (Figure 1 and Table 2; Gummersheimer and Danthi, 2020).

Non-turreted reoviruses such as rotaviruses and Bluetongue virus (BTV, *Orbivirus* genus) are triple-layered. After removal of the outer layer, the core particles that enter the cytoplasm of infected cells are double-layered and consist of the inner shells that are completely coated with a layer of trimers with *T* = 13/icosahedral symmetry (VP6 trimers for rotavirus (Settembre et al., 2011); VP7 trimers for BTV (Zhang et al., 2016; Figure 2 and Table 3).

However, a layer of trimers with *T* = 13/icosahedral symmetry also occurs in turreted reoviruses (e.g., μ1 in orthoreovirus and P9 in oryzavirus (Coulibaly et al., 2005; Miyazaki et al., 2008). Moreover, the layer of trimers has often been implicated in cell membrane penetration, as observed for μ1 in orthoreovirus infections of mammalian cells but also for trimers in infections of BTV of dipteran cells and plant reoviruses of hemipteran cells (see sections 7 and 8).

Regardless of whether core particles are double- or single-layered, the outer capsid layers of orthoreoviruses, rotaviruses and BTV contain the host receptor-binding proteins and the machinery for the penetration of the cellular membrane for their efficient delivery in the cytoplasm during their infection of mammalian cells (Herrmann et al., 2021; Roth et al., 2021). During this process, the outer layer proteins are discarded in the endosomes or at the cell surface while the core particles enter the cytoplasm as intact entities that initiate transcription without further disassembly (Lourenco and Roy, 2011; Patel and Roy, 2014). However, the outer layer proteins in the different groups of reoviruses that infect mammalian cells are largely non-homologous since they represent unique mechanisms by which each virus interacts with and enters the host cell. On the other hand, as will be outlined in more detail below, reoviruses that infect dipteran and hemipteran insect cells (arbo-reoviruses and plant viruses) have a less elaborate structure and the penetration mechanism may be more conserved because of its reliance on the presence of the layer of trimers.

## Molecular Mechanism of Cellular Entry by Orthoreoviruses in Mammalian Cells

Conform to the original indication of reoviruses as “respiratory enteric orphan viruses,” orthoreoviruses cause mild intestinal (and respiratory) diseases in many mammalian species, including humans. Orthoreoviruses are distinguished as three major serotypes, represented by the strains type 1 Lang (T1L), type 2 Jones (T2J) and type 3 Dearing (T3D; Chappell et al., 2002). The three strains differ primarily in the sequence of the σ1 spike protein that influences viral tropism and pattern of disease (Duncan et al., 1990).

Mechanistic studies of orthoreovirus entry, replication and release from cells have been greatly facilitated by the availability of primary and immortalized cell lines that support infection *in vitro* (Roth et al., 2021). Clathrin-mediated endocytosis is considered the most important uptake route in both polarized and non-polarized cells (Maginnis et al., 2008; Boulant et al., 2013) although other entry pathways were observed such as caveolar endocytosis (Schulz et al., 2012) and macropinocytosis (in neurons; Aravamudhan et al., 2020).

Virus infection involves a series of successive interactions with the host cell of which the steps include binding to the cell surface, intracellular trafficking, membrane penetration, activation of cell signaling and apoptosis. It starts with the binding to primary attachment factors that concentrate the virions on the cell surface and subsequently stimulate engagement with secondary receptors before internalization (Grove and Marsh, 2013). In the case of reoviruses, the result will be the delivery of a core particle in the cytoplasm that contains the intact dsRNA genome and is transcriptionally competent (Roth et al., 2021).

### Cell Attachment

Attachment factors that mediate low-affinity interactions with orthoreoviruses are sialylated glycans on the cell surface that interact with the σ1 spike protein (Reiter et al., 2011; Reiss et al., 2012; Figure 1 and Table 2). Anchored by interactions on top of the turrets, the σ1 spike protein extends from the surface of the virions and forms homotrimers that assemble to a lollipop shape consisting of a base, a long fibrous tail, a neck and a globular head (Chappell et al., 2002). Spike proteins from different serotypes have unique binding sites that interact with distinct sialylated glycan structures which contribute to the different cellular tropism of the strains (Barton et al., 2001). Although not absolutely necessary for productive infection, interactions with sialylated glycans increase the adhesion to the cell surface and promote the binding to proteinaceous receptors.

Junctional adhesion molecule A (JAM-A) was identified as a high-affinity attachment factor for all orthoreovirus serotypes (Barton et al., 2001). JAM-A is a member of the immunoglobulin family that regulates tight junctions in epithelia but is also expressed in orthoreovirus-permissive cell lines. A second entry receptor is the Nogo receptor 1 (NgR1), a glycosylphosphatidylinositol (GPI)-anchored protein with leucine-rich repeats (LRR) that is expressed on the surface of neurons and considered responsible for reovirus tropism in the central nervous system (Konopka-Anstadt et al., 2014). JAM-A and NgR1 interact with different capsid components in virions. Crystal structures show that the membrane-distal immunoglobulin-like D1 domain of JAM-A engages a JAM-A binding site in the lower part of the head domain of the σ1 spike protein, that is conserved between T1 and T3 serotypes (Stettner et al., 2015), while the viral component that interacts with NgR1 is likely the outer layer capsid protein σ3 (Roth et al., 2021). Interestingly, NF-κB signaling and apoptosis can be induced via interaction of σ1 with JAM-A (Barton et al., 2001) but several other early events during infection were also found to be implicated (Connolly and Dermody, 2002; Danthi et al., 2013) and the events that activate NF-κB signaling and stimulate apoptosis following orthoreovirus infection remain incompletely understood (Danthi et al., 2013).

### Cellular Internalization

Binding to sialylated glycans or the JAM-A receptor is not sufficient for cellular uptake which requires secondary interaction with integrins that will recruit the endocytic machinery (Maginnis et al., 2008). Binding to the β1 integrin internalization receptor may occur through integrin-binding motifs (RGD and KGE) present in the turret protein λ2 (Maginnis et al., 2006). After internalization, virions will traffic into the endosomal compartment (Boulant et al., 2013). During this process, acidification of the endosome occurs and the outer layers of the virions are processed by cathepsin B and cathepsin L proteases to generate infectious subviral particles (ISVPs; Ebert et al., 2002). ISVPs differ from virions by the removal of the clamp protein of the outer layer, σ3, the proteolytic processing of the underlying protein μ1 and a conformational change in the protein σ1 from a flexible conformation to the appearance of fiber-like spikes projecting from the turrets (Liemann et al., 2002).

The penetration protein μ1 (that forms the *T* = 13/icosahedrical layer of trimers) (Figure 1 and Table 2) undergoes two distinct proteolytic processing events. The first occurs early during virion assembly, is autocatalytic and involves the production of a small N-terminal fragment that is myristoylated (μ1N, 4 kDa) and a large C-terminal fragment μ1C (72 kDa; Nibert and Fields, 1992). Both fragments remain associated with the released virions. During IVSP formation in the endosomes or in the intestinal lumen (see below) μ1C undergoes additional proteolytic cleavages to large N-terminal fragments (60–70 kDa; μ1δ and δ) and a small C-terminal fragment (φ; 13 kDa; Nibert and Fields, 1992). The mechanisms that underlie membrane penetration are not completely understood but involve further transformation of IVSPs to IVSP^∗^s that includes the loss of σ1, a structural rearrangement in μ1C (or its fragments) that exposes hydrophobic residues and release of myristoylated μ1N to form membrane pores (with the φ fragment as possible chaperone) (Chandran et al., 2002; Zhang et al., 2006; Danthi et al., 2013). An anion-binding site in the μ1C fragment may promote its binding to phospholipid head groups on the cellular membrane (Liemann et al., 2002).

### Endosomal Trafficking

After internalization, localization of reovirus particles to early and late endosomes but not to recycling endosomes (characterized by distinct Rab GTPases) is associated with productive infection (Mainou and Dermody, 2012). Interestingly, activation of Src kinase signaling (by unknown mechanism) is necessary for appropriate sorting of reovirus particles in the endosomal compartment and to avoid their targeting to the lysosomes (Mainou and Dermody, 2011). However, interferon-inducible transmembrane protein 3 (IFITM3), involved in antiviral defense pathways, restricts the entry of orthoreovirus at the late endosomes by interfering with proteolytic processing of the μ1 penetration protein (Anafu et al., 2013). Advanced 3D correlative cryo-structured illumination fluorescence and soft X-ray microscopy techniques show the release of orthoreoviral particles at Rab7-positive late endosomes after 1–2 h after infection (Kounatidis et al., 2020). Furthermore, early small vesicles with viral load merge to larger vesicles and develop into multivesicular bodies that contain remnants of the reovirus outer capsids. Vesicles at the site of viral egress remain structurally intact indicating rapid closure of membrane pores following passage of the core particles (Kounatidis et al., 2020).

### Infection of Polarized Epithelia

With respect to the infection of the highly polarized intestinal epithelium *in vivo* (where initial infection occurs by the oral-fetal route before the virus spreads to internal organs), orthoreovirus particles are known to undergo proteolytic processing in the intestinal lumen (Amerongen et al., 1994). Chymotrypsin and trypsin proteases degrade the outer capsid protein σ3 and cleave the underlying μ1 protein into membrane-penetrating moieties to generate ISVPs (Mohamed et al., 2015). Because of the thick glycocalyx that covers the brush border of absorptive enterocytes, their apical surfaces are not accessible to virions or ISVPs. Instead, orthoreoviruses and other pathogens pass more easily the intestinal barrier in regions that contain organized lymphoid follicular tissues (such as Peyer’s patches). Follicle-associated epithelia contain specialized microfold cells (M cells) that are specialized to transport luminal material (antigens) to the underlying mucosa and that have only a thin coat of glycoproteins which makes them accessible to binding by ISVPs (Helander et al., 2003). After entry into the intestinal mucosa via M cell transcytosis, orthoreoviruses will be able to adhere to the basolateral membranes of the absorptive enterocytes (with the highest levels of expression of the JAM-A receptor for viral entry) as well as to phagocytic cells (macrophages) (Amerongen et al., 1994; Helander et al., 2003; Mohamed et al., 2015). In the intestine, ISVPs are more infectious than unprocessed virions, which indicates an adaptation to the harsh environment of the intestinal lumen.

In *in vitro* models of polarized epithelial cells, ISVPs establish infections much more readily than virions since they do not require kinase signaling, endosomal acidification or cathepsin proteases (Boulant et al., 2013; Stanifer et al., 2016). ISVPs enter the cytosol of polarized epithelia at an earlier stage than late lysosomes which prevent their interaction with innate immune factors such as IFITM3. In the T84 cell line, a model for human intestinal epithelial cells, ISVPs induce transforming growth factor (TGF) β signaling to prevent the induction of apoptosis and to increase the efficiency of virus infection (Stanifer et al., 2016). These observations indicate that infection of intestinal epithelium by ISVPs *in vivo* can differ significantly from infection of permanent cell lines by virions.

### Efficiency of Infection and Availability of Reverse Genetics Systems

Because failure can occur at many steps in a complex cell entry process, orthoreovirus infections are characterized by high particle-to-plaque forming unit (pfu) ratios (100–1000; Danthi et al., 2008). The protein μ1 of the outer capsid layer is considered an important determinant of the infectious dose because it determines the efficiency of penetration of the cellular membrane and regulates the overall stability of the capsid to prevent the release of dsRNA and its interaction with immune sensors (Danthi et al., 2008). Finally, *in vitro* reverse genetics systems for orthoreoviruses have been established that allow development of recombinant reoviruses for basic research and for clinical studies (Komoto et al., 2014).

## Molecular Mechanism of Cellular Entry by Rotaviruses in Mammalian Cells

Rotaviruses exhibit a very distinct tropism and infect primarily differentiated enterocytes at the top of the intestinal villi in the jejunum of the small intestine (Guerrero et al., 2000). As is the case for orthoreoviruses, a major achievement was the establishment of cell culture models, in which simian rotaviruses (e.g., Rhesus rotavirus) could efficiently infect polarized cell lines of renal or intestinal epithelial origin (Estes et al., 1979; Suzuki, 2019). More recently, the technology of human intestinal enteroids has allowed the study of the pathophysiology of human rotaviruses (Saxena et al., 2015).

### Cell Attachment

For the virus to become infectious, the outer capsid (spike) protein VP4 needs to be proteolytically processed by trypsin-like proteases in the intestinal lumen (Pesavento et al., 2006; Figure 2 and Table 3). Proteolysis of the spike protein VP4 results in two fragments, VP8^∗^ and VP5^∗^, that remain associated with virions. The N-terminal VP8^∗^ fragment is involved in the initial attachment through interactions with glycans on the cell surface (Arias and López, 2021). Binding to glycans is considered critical for tissue tropism and host specificity and animal rotaviruses mainly interact with sialoglycans while human rotaviruses preferentially recognize polymorphic histoblood group antigens (Hu et al., 2018). The large diversity in VP4 proteins forms the basis for the classification of the P genotypes of rotaviruses which is reflected in the binding of VP8 to different glycans (Liu et al., 2017).

On the other hand, a proteinaceous receptor that is essential for rotavirus entry so far has not been identified (Arias and López, 2021). Interactions between integrin α2β1 and VP5^∗^ and of integrins αvβ3 and αxβ2 with the other outer capsid protein VP7 have been demonstrated for Rhesus rotavirus (Guerrero et al., 2000; Graham et al., 2003) but structural studies indicate that the accessibility of the predicted integrin-binding motifs (DGE in VP5^∗^, GPR in VP7) depends on proteolytic processing and conformational changes at later steps in the uptake process (Abdelhakim et al., 2014).

### Cellular Internalization

For cellular entry, rotaviruses share common requirements of dynamin, the presence of cholesterol on the cell surface and heat-shock cognate 71-kDa protein (HSC70) but they differ in their use of internalization pathways: while the entry route of Rhesus rotavirus is clathrin- and caveolin-independent, most other rotaviruses use clathrin-dependent endocytosis (Gutierrez et al., 2010). For all rotaviruses, however, the major determinant for cell entry is the spike protein VP4 which defines the interaction with cellular factors downstream of the initial attachments to glycans (Díaz-Salinas et al., 2013).

In human epithelial cells and enteroids, evidence for a specific (unidentified) internalization receptor that interacts with VP8^∗^ was revealed by the use of a panel of neutralization antibodies (Feng et al., 2019). In highly polarized cells, binding of rotavirus virions to cellular receptors activates RhoA/ROCK/Myosin Light chain signaling, resulting in a reorganization of the cytoskeleton and disruption of tight junction integrity. Through this process, virions acquire access to the basolateral membrane where binding can occur with putative co-receptors such as JAM-A and possibly occludin (Soliman et al., 2018a).

Direct cell membrane penetration of rotaviral particles, in the absence of endocytosis, has also been observed (Suzuki, 2019). Using infection of the green monkey kidney cell line BSC-1 by Rhesus rotavirus as a model, the interaction with the host cells of “triple-layered particles” (i.e., virions), that were differentially fluorescently labeled on outer layer and inner layer capsid proteins, was monitored by live cell imaging (Abdelhakim et al., 2014). The delivery of “double-layered particles” (DLPs) into the cytoplasm occurs within 10 min and electron cryotomography images show engulfment of viral particles by tight-fitting membranes to form vesicles that are too narrow for being endosomes. The results are interpreted that the virions drive the penetration of DLPs through the plasma membrane following membrane-bound conformational changes in the outer layer capsid that disrupt the lipid bilayer (Abdelhakim et al., 2014).

### Endosomal Trafficking

In cases when simian rotaviruses enter the cells by (clathrin-dependent or -independent) endocytosis, passage is required through early endosomes while escape of DLPs occurs at the stage of maturing endosomes that generate intraluminal vesicles to become multivesicular bodies (as “early-penetrating” rotaviruses; Silva-Ayala et al., 2013). However, other rotaviruses are characterized as “late-penetrating” since they require progression to late endosomes (Díaz-Salinas et al., 2014). Furthermore, productive infection by late-penetrating virions requires the recycling of cholesterol between endoplasmic reticulum and late endosomes (Civra et al., 2018) and the function of the cation-dependent mannose-6-phosphate receptor for transport of cellular factors from the *trans*-Golgi network to the late endosomes, as well as the cathepsins B, L and S (Díaz-Salinas et al., 2014). Late-penetrating rotaviral strains also activate extracellular signal-regulated kinase (ERK) and the phosphoinositide 3-kinase (PI3K)-AKT pathways for acidification of late endosomes and activation of cathepsin proteases to facilitate uncoating of virions (Soliman et al., 2018b). Differences in entry route by early- and late-penetrating rotaviruses are associated with the spike protein VP4 (Díaz-Salinas et al., 2014).

### Detailed Description of Structural Rearrangements in the Spike Protein for Membrane Penetration

In the processed spike protein VP4, the main function of VP8^∗^ consists of the attachment to the cell surface, while VP5^∗^ provides the means to perforate the cellular or vesicular membrane (Herrmann et al., 2021). VP4 is a trimer but structurally rearranges on the surface of the virions in an asymmetric configuration that consists of foot, stalk, body and head regions (Rodríguez et al., 2014). Although treatment with trypsin results in the cleavage of the spike protein VP4 into VP5^∗^ and VP8^∗^ and primes the virions for efficient infectivity, it does not change the overall structure of the spikes indicating that cleavage is only necessary to allow the conformational changes that drive virus entry (Rodríguez et al., 2014). The activation process of VP4 therefore resembles that of membrane fusion proteins of enveloped viruses such as Semliki forest virus and dengue virus (Dormitzer et al., 2004).

The foot domain of VP4 is anchored in depressions of the VP6 trimer layer of the DLPs (with *T* = 13/icosahedral symmetry; Figure 2 and Table 3) and is clamped by the other outer capsid layer VP7 glycoprotein (Settembre et al., 2011). VP7 forms Ca^2+^-stabilized trimers that dissociate following the loss of Ca^2+^, for instance during membrane permeabilization of the endosome, which results in the release of VP8^∗^ and VP5^∗^ and uncoating of the virion (Salgado et al., 2018).

During “loose” contacts with the cell membrane, the spike protein displays an extended asymmetric configuration in which the head and body are formed by VP8^∗^ and VP5^∗^ domains, respectively, of two VP4 spike proteins; the VP5^∗^ domain of the third VP4 constitutes the stalk through unique interactions with VP7; and the third VP8^∗^ domain is either lost or sequestered at the foot domain (Rodríguez et al., 2014). After initial interactions of the glycan-interacting lectin domains of VP8^∗^ with the cell surface, VP4 undergoes a transition from an upright conformation to a so-called “reverse” formation: the third VP5^∗^ of the stalk flips outward and forms a trimer of β-barrel domains with the two VP5^∗^ domains of the body, and zippering of a three-strand α-helical coiled-coil at the center causes the unfolding and outward projection of the foot domain (Herrmann et al., 2021). “Tight” contacts with the cell membrane may be triggered by lateral movements of the lectin domains that will expose the hydrophobic loops of the two β-barrel domains of VP5^∗^ in the body of the spike; after adopting the reverse conformation, hydrophobic regions of the protruded foot domain can insert in the membrane and likely cause larger scale disruptions (perforations) for delivery of the DLPs into the cytoplasm (Herrmann et al., 2021).

### Efficiency of Infection and Availability of Reverse Genetics Systems

Rotavirus reverse genetics systems have become available for basic studies, generation of reporter strains and development of virus-based vaccines (Kanai and Kobayashi, 2021). Reflecting rotavirus entry as a multistep process, measurements of ratios of infectious particles to physical particles yielded low values that ranged from 1/100 to less than 1/10.000, depending on the strain (Mendez et al., 1999). When fluorescently labeled single particles were followed during the infection process and viral RNA synthesis was checked by *in situ* hybridization, efficiency estimations were 10% for cell attachment, 20–50% for uncoating and 10–15% for RNA synthesis, giving a ratio of 1/250 to 1/500 (Salgado et al., 2017).

## Molecular Mechanism of Cellular Entry by BTV (Genus: *Orbivirus*) in Mammalian and Insect Cells

Bluetongue virus is an arbovirus that is transmitted between ruminants and competent dipteran insect vectors (Ceratopogonidae, midges) (Belbis et al., 2017). Most severe cases of disease were reported in sheep although disease outbreaks also occurred in cattle that were associated with particular BTV serotypes (Martinelle et al., 2018). However, BTV can also be transmitted through the semen and can cross the transplacental barrier (Belbis et al., 2017). In contrast to orthoreoviruses and rotaviruses, that enter the (vertebrate) organism through infection of epithelia, BTV and related orbiviral species (e.g., African horse sickness virus and epizootic hemorrhagic disease virus) are horizontally transmitted by the bites of hematophagous female midges of the *Culicoides* genus (Diptera: Ceratopogonidae) (Mertens et al., 2005). After intradermal inoculation, BTV primarily infects different mammalian blood cell types (lymphocytes, monocytes, dendritic cells and macrophages) before dissemination to secondary infection sites (lungs, spleen, and lymph nodes) (Mclachlan et al., 2009).

Many different mammalian cell lines are susceptible to BTV infection and show cytopathic effects (as acute pathogenic infections; Wechsler and McHolland, 1988). After the isolation from animal tissues, BTV is usually amplified in embryonating chicken eggs before passage to cultured cells such as African green monkey kidney (Vero) and baby hamster kidney (BHK-21) cells (Clavijo et al., 2000).

When *Culicoides* midges feed on an infectious blood meal, BTV encounter the typical barriers for infecting a dipteran insect vector: infection and proliferation of midgut cells, midgut escape, dissemination through the body and infection of the salivary glands (Mills et al., 2017). BTV can also replicate in dipteran insect cell lines such as mosquito C6/36 and Aag2 cells and midge Kc cells, although only minor cytopathic effects are observed consistent with persistent infections (Wechsler and McHolland, 1988; Schnettler et al., 2013).

### Infection of Mammalian Cells: Entry Routes

Bluetongue virus can infect a wide variety of cell types in mammalian hosts such as endothelial cells, T cells, dendritic cells and a range of leukocytes and it is therefore not surprising that it can employ different cell entry routes. In HeLa and Vero cells, BTV enters through clathrin-mediated endocytosis and exits the endosomal pathway at the early or late endosomes (Forzan et al., 2007). In BHK-1 cells, on the other hand, macropinocytosis is implicated as an uptake mechanism and the virions are delivered to late endosomes without passage through early endosomes (Gold et al., 2010; Stevens et al., 2019). By contrast, caveolae- or lipid raft-mediated mechanisms or direct cell membrane penetration are probably not used by BTV, in contrast to orthoreoviruses and rotaviruses (Roy and Noad, 2006; Gold et al., 2010).

For both clathrin-mediated endocytosis and macropinocytosis, however, (endosomal) acidification is an essential requirement for successful BTV infection. Detachment of VP2 (spike) trimers from the virions is observed at pH 6.0–6.5, while the conformational changes necessary for membrane penetration by VP5 occur at pH 5.5–6.0, which is likely caused by the build-up of repulsive electrostatic forces within and between capsid proteins (Konevtsova et al., 2019). Visualization of fluorescent viral particles during cell entry indeed revealed shedding of VP2 in early endosomes while VP5 could traffic to late endosomes (Du et al., 2014), where membrane penetration is predicted to occur. Membrane penetration is facilitated by the particular composition of lipids in late endosomes that promote membrane fluidity (Patel et al., 2016).

### Infection of Mammalian Cells: Triple-Layered Particles

In contrast to other reoviruses, the outer shell of the BTV virions, which is composed of VP2 and VP5 trimers, is highly unstable (Figure 2 and Table 3; Zhang et al., 2010a; Konevtsova et al., 2019) and infectivity of virions is lost in mildly acidic conditions or after treatment with detergents and lipid solvents (Hassan et al., 2001). Actually, the virion structure of BTV also appears more fragile at the inner levels of its organization. When expressed by strong baculovirus promoters in lepidopteran insect cells, the inner shell protein VP3 does not assemble spontaneously in “subcore”-like particles but requires co-expression of VP7 to form double-layered core-like particles (Figure 2 and Table 3; Hewat et al., 1992). However, the spaces of the VP7 trimers in the second layer are significantly smaller than the surfaces that need to be occupied and the second shell therefore needs to be reinforced structurally by interactions with capsid proteins of the third layer (Konevtsova et al., 2019). As a first step in the formation of the triple-layered particle, VP5 trimers become deposited in the spaces between the VP7 trimers in the second shell which is subsequently followed by the addition of the VP2 trimers that also link with four VP7 trimers from the underlying second shell (Mohl and Roy, 2014).

### Infection of Mammalian Cells: Cell Attachment

Of the two outer layer capsid proteins, VP2 is the component which mediates the attachment to the cell surface and triggers internalization through clathrin-mediated endocytosis (Roy, 2005). On the virion surface, the 110 kDa VP2 (Figure 2 and Table 3) assembles as a trimer to form a sail-shaped protruding triskelion spike-like structure (Zhang et al., 2010a). Each VP2 monomer is divided in 4 domains, i.e., a tip domain, ideally located for interaction with the cell surface (through an unknown receptor), a body domain, a hairpin, and a hub domain, that has a similar fold as the sialic acid-binding domain of VP8^∗^ of the rotavirus spike (Zhang et al., 2016). Besides mediating cell attachment, binding to sialic acids may also promote adsorption to erythrocytes and stimulate uptake by the *Culicoides* vector during blood feeding (Bhattacharya and Roy, 2010). In addition, VP2 contains a zinc finger motif at the interface of the hub domain and the body domain, which plays a role in the pH-sensing mechanism that triggers the detachment of VP2 from the virion during cell entry in the early endosome (Zhang et al., 2016).

### Infection of Mammalian Cells: Cellular Internalization

Interspersed with the VP2 triskelion on the virion surface are trimers of VP5 (Figure 2 and Table 3) that form globular complexes and are involved in membrane penetration (Bhattacharya and Roy, 2010; Zhang et al., 2016). Each VP5 monomer consists of three domains designated as “dagger,” “unfurling,” and “anchoring.” After dissociation of VP2 in the early endosome, VP5, that remains attached to the virion, forms a protruding barb-like filamentous structure through refolding of the dagger and unfurling domains, corresponding to what is described as a “flower-like” opening of the VP5 trimer (Zhang et al., 2016). Protonation of histidine residues at low pH are thought to play a major role in the conformational changes that expose the dagger domain for membrane interaction. Similar to the mechanism observed for VP5^∗^ of rotavirus, initial contacts with the endosomal membrane are also predicted to launch further conformational changes in VP5 of BTV with additional hydrophobic helices unfurling and perforating the membrane layer. During this process, VP5 would also detach and the core particles be delivered in the cytoplasm (Bhattacharya and Roy, 2010; Zhang et al., 2010a, 2016). In the proposed mechanism proteolytic activation is not required, in contrast to other viral fusion proteins.

### Infection of Mammalian Cells: Signaling Pathways

Relatively little is known regarding the activation of cellular pathways during early BTV infection of mammalian cells. Extracellular treatment with virions or a combination of the two outer capsid proteins VP2 and VP5 results in the activation of apoptosis, which was dependent on endosomal acidification (Mortola et al., 2004). Activation of NFκ-B during BTV infection was also observed but shown to be involved in an antiviral response (and neutralized by viral proteins) and not in the induction of apoptosis (Stewart and Roy, 2010). In addition, induction of MAPK/ERK by BTV (but not other orbiviruses) was demonstrated as a relatively late event during infection that requires production of the viral protein NS3 and functions to stimulate viral replication and spreading (Kundlacz et al., 2019).

### Infection of Insect Cells: Processing to ISVPs

In competent midge vectors, BTV is taken up by the midgut following feeding on an infectious blood meal. Electron microscopy images show adsorption of BTV to erythrocytes and their entry into midgut epithelial cells through the microvilli (Sieburth et al., 1991). Because of their exposure to the midgut lumen, it is expected that BTV virions undergo proteolytic processing before cell entry. When purified BTV virions are treated with proteases such as trypsin or chymotrypsin, ISVPs are produced that are characterized by a loss of NS2 (a non-structural protein from the virogenic stroma) from the virion and cleavage of VP2 of which the fragments remain associated with the particles (Mertens et al., 1996). Further treatment with cations results in (double-layered) core particles that consist of the VP3 shell and the intermediate layer of VP7 trimers (that encapsulate the dsRNA genome and associated replication proteins).

Infectious subviral particles showed much higher infectivity in midge cells than in mammalian cells and it was estimated that prior treatment by proteases (which could occur in the gut lumen but also (prior to uptake by the insect) in mammalian blood) could raise infectivity for competent midge vectors by 200–2000 fold (Mertens et al., 1996). These data indicate that proteolytic processing of virus particles plays a significant role during the natural infection of the midgut epithelium of midge vectors by BTV.

### Infection of Insect Cells: Uptake of Core Particles

Furthermore, core particles retained similar infectivity as virions in *Culicoides* cells, in contrast to mammalian cells, where removal of the outer capsid proteins reduces significantly the infectivity (Mertens et al., 1996). In core particles, the outer layer is formed by VP7 trimers (Figure 2 and Table 3). Interestingly, an accessible RGD tripeptide motif was identified in VP7 that is responsible for binding to *Culicoides* cells, indicating that the entry mechanism of core particles in midge cells involves interaction with integrins (Tan et al., 2001). Thus, infection of *Culicoides* midges may use multiple attachment and entry mechanisms, involving VP2/VP5 (for intact virions and ISVPs) or VP7 (for core particles) and interaction with distinct cell receptors. A recent study identified decreased binding of VP2 to the cell surface as one of the mechanisms by which the BTV-26 variant fails to infect and replicate in *Culicoides* cells (Guimerà Busquets et al., 2021).

### Infection of Insect Cells: Signaling Pathways

Uptake of BTV by insect cells also involves clathrin-mediated endocytosis (Mills et al., 2017) and transcriptome analysis of infected mosquito cells indicated differential expression of mRNAs related with endocytosis and the insulin, MAPK, Hippo and Jak/STAT pathways (Du et al., 2019). In infected *Culicoides* and mosquito cells, the antiviral RNAi pathway is activated which was mainly characterized by the production of viral siRNAs (Schnettler et al., 2013).

### Efficiency of Infection and Availability of Reverse Genetics Systems

Reverse genetics systems for BTV were developed based on *in vitro* transcribed RNA (Boyce et al., 2008) and expression plasmids (Pretorius et al., 2015) with applications in nanotechnology and vaccine development (Roy, 2020; Thuenemann et al., 2021). The particle-to-pfu ratio for BTV infection of mammalian cells is reported to be very low (1–3; Wu et al., 2019; Labadie and Roy, 2020) compared to other viruses, illustrating high infection efficiency. In addition, it has recently been reported that BTV virions can be released in lysosome-derived extracellular vesicles that are even more efficient than free viruses to initiate infections (although they use another uptake mechanism; Labadie and Roy, 2020).

## Molecular Mechanism of Cellular Entry by Plant Reoviruses in Insect Cells

Plant reoviruses are transmitted by hemipteran insect vectors in a persistent circulatory propagative manner, which means that viruses cross the midgut barrier and replicate in internal organs (Nault, 1997; Hogenhout et al., 2008; Kolliopoulou et al., 2020). Most studies have focused on reoviruses that infect and cause disease in rice such as Rice dwarf virus (RDV; *Phytoreovirus* genus), Rice black-streaked dwarf virus and Southern rice black-streaked dwarf virus (RBSDV and SRBSDV; *Fijivirus* genus) and Rice ragged stunt virus (RRSV; *Oryzavirus* genus) (Wei and Li, 2016). Of these genera, *Fijivirus* and *Oryzavirus* belong to the turreted *Spinareovirinae* subfamily; however, the best-studied species is RDV (*Phytoreovirus*) which is non-turreted (*Sedoreovirinae* subfamily). Plant reoviruses are associated for transmission with particular hemipteran insect vector families: while reoviruses of the *Phytoreovirus* genus are transmitted by leafhoppers (Cicadellidae), reoviruses of *Fijivirus* and *Oryzavirus* genera are vectored by planthoppers (Delphacidae) (Kolliopoulou et al., 2020).

### Molecular Mechanism of Cell Entry by Rice Dwarf Virus (Genus *Phytoreovirus*)

Rice dwarf virus forms double-layered particles of 70 nm that are equivalent to the core particles of BTV (Nakagawa et al., 2003). The inner shell consists of 120 copies of P3 core capsid protein (114 kDa) which is covered with a *T* = 13/icosahedral layer of 260 trimers of P8 (46 kDa) (Figure 2 and Table 3; Omura and Yan, 1999). The outer layer of the virion also contains the minor capsid protein P2 (127 kDa) which is essential for infection of the hemipteran vector (Zhong et al., 2003). Baculovirus-mediated expression of P3 indeed resulted in the formation of single-layered particles while co-expression with P8 generated double-layered particles (Hagiwara et al., 2003). Interestingly, P7, a nucleic acid-binding protein that can bind genomic dsRNAs, was also observed to be recruited inside the inner shell during baculovirus-mediated expression.

Particles that only contain P8 in the second layer can infect leafhoppers by direct injection in the hemolymph while P2 is additionally required for infection after feeding (Nakagawa et al., 2003). As the homolog of μ1 protein of orthoreoviruses, it can be inferred that P8 plays a role in membrane penetration during infection of hemipteran vector cells (Omura and Yan, 1999). On the other hand, P2 contains structural features that are similar to type I membrane fusion proteins from enveloped viruses such as an N-terminal hydrophobic peptide, heptad repeats and a C-terminal transmembrane domain. Conform to a role in membrane destabilization, P2 can trigger cell-cell fusion after exposure of expressing cells to low pH (Zhou et al., 2007), as was also observed for the penetration protein VP5^∗^ of rotavirus (Forzan et al., 2004). Since RDV is taken up by clathrin-mediated endocytosis in “vector cell monolayers” (continuous cell lines derived from leafhoppers; Omura and Kimura, 1994) acidification of the endosomes is considered as the trigger for the uptake of the virus (Wei et al., 2007).

P2 proteins are L-shaped and are anchored on the surface of the virion in pores formed by two P8 trimers at 5-fold axes in the icosahedral capsids (Miyazaki et al., 2016). The long axes of P2 are proposed to protrude from the surface of the virions for a distance of 15 nm to make contact with the host cell membrane. In contrast to fusion proteins of enveloped viruses, P2 does not require proteolytic processing to expose the fusion peptide at the N-terminus. Since P2 is loosely attached (10–30 copies per virion) it could insert in the endosomal membrane and cause its destabilization (Zhou et al., 2007).

In the alimentary canal, RDV initially infects the filter chamber from where it spreads to other epithelia in the gut, the visceral muscles and the internal tissues (Chen et al., 2011). Viral spread between cells in vector cell monolayers and *in vivo* is promoted by the non-structural protein Pns10 that assembles into helical tubular structures that contain virions (Chen et al., 2012). Interaction with the actin cytoskeleton can provide a propulsive force for the spread of virions between cells, through the basal lamina or into the gut lumen (Jia et al., 2018). Both P2 and Pns10 are only required for infection of the hemipteran vector and not for infection of plants. Plant viruses are believed to enter plants through wounding (e.g., during feeding by viruliferous vectors) and to spread from cell-to-cell in plants through plasmodesmata (Miyazaki et al., 2013).

In the related phytoreovirus Rice gall dwarf virus (RGDV), fibrillary structures formed by the non-structural protein Pns11 target the mitochondria to trigger apoptosis (Chen et al., 2019). Apoptosis and other cytopathological effects caused by virus replication are thought to promote the spread of the virus in the hemipteran vector. On the other hand, activation of the RNAi response in the hemipteran vectors is proposed to control replication of RGDV to limit damage and maintain the persistent character of phytoreovirus infections (Lan et al., 2016b).

### Molecular Mechanism of Cell Entry by Other Plant Viruses in Hemipteran Vectors

Other plant viruses of the genera *Oryzavirus* and *Fijivirus* that infect delphacid planthoppers are turreted reoviruses (subfamily *Spinareovirinae*) but their structure is less elaborate than the orthoreoviruses that infect mammals/vertebrates.

The structure of Rice ragged stunt virus (RRSV; *Oryzavirus*) reveals a core particle (assembled by 120 copies of P3 inner shell protein) that shows the 12 canonical turrets (each containing 5 copies of turret protein P2) but contains less clamp protein (120 copies of P8b in RRSV *versus* 150 copies of σ2 in mammalian orthoreovirus) (Figure 1 and Table 2; Miyazaki et al., 2008). Regarding the second layer, 60 peripheral trimers of P9 are present that only partially cover the core shell while 200 μ1 trimers are found in orthoreoviruses (Figure 1 and Table 2). In orthoreoviruses, trimers of μ1 play an important role in membrane penetration, as discussed above (Liemann et al., 2002). Interestingly, P9 (59 kDa) also plays an important role during infection of the hemipteran vector and a putative interacting protein (putative receptor) was identified in cell membranes of planthoppers (Zhou et al., 1999; Shao et al., 2003). By contrast, no “protection proteins” were identified in the outer layer of RRSV, *i.e.*, homologs of orthoreovirus σ3 that keep μ1 inactive and coat the surface of orthoreoviruses as σ3/μ3 complexes (Liemann et al., 2002; see section 5). Finally, no spike protein was identified in RRSV virions (Miyazaki et al., 2008); however a protein with regions of homology with the “A-spike” protein of cypovirus (to be discussed later) is encoded by segment 1 (P1, 138 kDa) and it is possible that the spike did not appear in the structure of RRSV virions because of its loss during the preparation of virions or its high mobility on the surface (Miyazaki et al., 2008). The putative function of the A-spike protein of cypoviruses and oryzaviruses will be discussed later.

Interestingly, virion structures of another turreted reovirus that belongs to the genus *Aquareovirus* and infects fish, also show a reduction in structural complexity compared to mammalian orthoreoviruses (Zhang et al., 2010b). There is a reduction in the number of clamp proteins (120 copies per virion) as in RRSV and the spike protein is absent (Jaafar et al., 2008); however aquareoviruses maintain a full outer layer of complexes of penetration protein trimers with protection protein trimers as observed in orthoreoviruses (Cheng et al., 2010).

Unfortunately, no structures were determined for virions of the *Fijivirus* genus but their two-layered organization could be constructed after treatment of purified virions with chemicals that remove the outer layers (Isogai et al., 1998; Wu et al., 2020). Thus, core particles of RBSDV consist of an inner shell (assembled by P2 protein, 141 kDa) and turrets (“B-spikes” composed of P3, 132 kDa) while no “clamps” were reported (P8 (68 kDa) is mentioned as minor core protein; Zhang et al., 2021). The outer layer contains trimers of P10 (63 kDa) and “A-spikes” (P4, 136 kDa). P10 (forming an incomplete *T* = 13/icosahedral layer of trimers) is considered important for invasion of hemipteran vectors and interacts with several host proteins (vesicle-associated membrane protein 7 (VAMP7), vesicle transport VSNARE protein (Vti1A), growth hormone-inducible transmembrane protein (Ghitm), that are proposed to play a role in the infection mechanism (Than et al., 2016). Because of their exposure on the surface of the virions, A-spikes of fijiviruses may be involved with the interaction with the cellular membrane in the hemipteran host but they do not share homology with putative spike proteins from oryzaviruses or cypoviruses (which is discussed in section 9) (Zhang et al., 2001; Liu et al., 2007; Wu et al., 2020).

Also for oryzaviruses and fijiviruses, *in vitro* cell cultures (“vector cell monolayers”) could be established as easily accessible models to study infection (Mao et al., 2013; Chen et al., 2014). However, most studies on infection were carried out with fijiviruses such as RBSDV and SRBSDV. As is the case for phytoreoviruses, RBSDV exploits virus-containing tubules (here based on P7-1) and their interaction with actin for spreading within the hemipteran vector (Jia et al., 2014). At high temperatures, SRBSDV hijacks the endoplasmic reticulum-associated degradation (ERAD) machinery and the heat-shock protein DnaJB11 to ensure efficient transmission (Yu et al., 2021). Activation of RNAi by fijivirus in their hemipteran vectors was also reported (Li et al., 2013; Lan et al., 2016a; de Haro et al., 2017). Interestingly, the RNAi response controls viral replication in the midgut and contributes to the vector’s competence for virus transmission (Lan et al., 2016a).

## Molecular Mechanism of Cell Entry by Cypoviruses in Lepidopteran Insects

Cypoviruses structurally are considered the simplest reoviruses since they lack the trimers that decorate the inner shell of other reoviruses and therefore correspond to core particles of orthoreoviruses (Yu et al., 2011; Li et al., 2017). The inner shell of the core particles is assembled by 120 copies of the “capsid shell protein” CSP or VP1 and is decorated with 12 turrets (each constructed with five “turret proteins” TP or VP3) and 120 copies of clamp proteins (“large protrusion protein” LPP or VP5) (Figure 1 and Table 2; Zhao et al., 2018). The single shell capsids of cypoviruses have a very high stability because of the unique “small protrusion” domain in VP1 that forms interactions with neighboring CSP and LPP molecules in the capsid (Yu et al., 2011; Ren et al., 2021).

### Formation of Polyhedra

A unique feature of cypoviruses is the inclusion of the virions in polyhedra for protection in the environment (Ji et al., 2015). Micrometer-sized polyhedra can contain several thousands of virions and are resistant to dehydration, freezing, enzymatic degradation and chemical treatments (Coulibaly et al., 2007). By contrast, polyhedra are efficiently dissolved at alkaline pH (as occurs in the midgut of lepidopteran larvae) which results in the release of the virions. The building block of the polyhedra is the polyhedrin protein (Figure 1 and Table 2) that has a similar structure and trimeric organization as μ1 in the second layer of orthoreovirus or VP7 in the middle layer of BTV. However, polyhedrin does not play a role in penetration such as μ1 but is involved in protection of virions in the environment. For that purpose, polyhedrin trimers do not organize on the surface of the shell as is the case for the trimers in other reoviruses, but form higher-order quaternary arrangements to become organized into a three-dimensional body-centered cubic lattice in which the virions are embedded (Coulibaly et al., 2007). Besides the dense packing of polyhedrin molecules, polyhedra also contain nucleotides (ATP, GTP, CTP) at strategic places, implying a role for them in controlling formation of the polyhedra in the cytoplasm.

Cypovirus virions contain “A-spikes” on the outer surface that project outwards from the top of the turrets (also called “B-spikes”) (in *Bombyx mori* CPV: Chen et al., 2011; Yu et al., 2011). When cypovirus virions that are embedded in polyhedra, are imaged with electron tomography, the A-spikes appear to make direct contact with the polyhedrin proteins in the polyhedra (Chen et al., 2011). However, polyhedrin appears to be a protein with promiscuous binding properties and contacts between polyhedrin and turret proteins have also been exploited for the recruitment of foreign proteins into polyhedron-based nanoparticles for biotechnological applications (Mori et al., 2007).

After the release of CPV virions from polyhedra in the highly alkaline environment of the midgut, the A-spikes are thought to play a role in the interaction with the cell surface and possibly also in membrane penetration (Yu et al., 2011). The A-spike consists of the large VP2 protein (140 kDa in CPV-1 or *B. mori* CPV) (Figure 1 and Table 2) in which no clear domains can be distinguished in the N-terminal three fourths of its length.

### A-Spikes With PPPDE Domains

**FIGURE 3:**
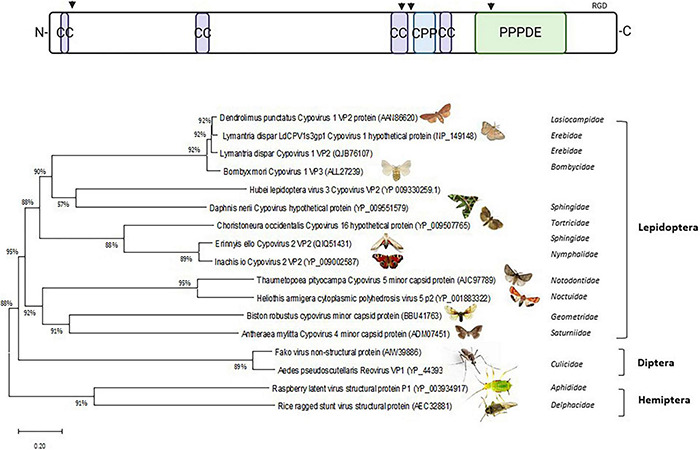
Phylogenetic analysis of VP2/A-spike of Cypovirus. **(Top)** Schematic of VP2 structure: CC, coiled-coil; CPP, cell-penetration peptide; PPPDE, Permuted Papain fold Peptidases of DsRNA viruses and Eukaryotes. Putative furin and cathepsin proteolytic sites are indicated with arrowheads. RGD is an integrin-interactive motif. Created with BioRender.com. **(Bottom)** Phylogenetic tree. To search for VP2 of BmCPV1 or Cypovirus 1 (Accession number: ALL27239) homologous proteins in other insect species a protein–protein BLAST search (BLASTp) was performed (https://blast.ncbi.nlm.nih.gov/). The BLAST searches ran against non-redundant protein sequences using the Blosum 62 matrix (Henikoff and Henikoff, 1992). Only the best hits were used with the lowest *E*-values (one for each organism). Phylogenetic trees and P-Distances were constructed using the neighbor-joining method (Saitou and Nei, 1987) with MEGA5 software (Tamura et al., 2011). Bootstrapping (Felsenstein, 1985) was used to estimate the reliability of phylogenetic reconstructions (1000 replicates).

Interestingly, VP2 proteins of cypoviruses are characterized by a “Permuted Papain fold Peptidases of DsRNA viruses and Eukaryotes” (PPPDE) domain (Iyer et al., 2004) at their C-termini. This domain is also present at the C-termini of similar proteins of reoviruses that infect mosquitoes (genus *Dinovernavirus*) (Attoui et al., 2005; Auguste et al., 2015) and those of plant reoviruses that are transmitted in a persistent circulative manner by planthoppers (RRSV, genus *Oryzavirus*; discussed in section 8) or aphids (raspberry latent virus; Quito-Avila et al., 2011, 2012; Figure 3 and Supplementary Figure 1). PPPDE domains function as cysteine isopeptidases with a papain-like fold and are predicted to have de-ubiquitinating and de-SUMOylating activities (Shin et al., 2012; Xie et al., 2017). The catalytic dyads of the PPPDE domains in VP2 of cypovirus and its homologs are intact and protease activity of PPPDE could be involved in the processing of viral polyproteins, capsid maturation and cleavage of host proteins (Iyer et al., 2004). In addition, the PPPDE domain could play a role in host-virus interactions by regulating cellular functions such as vesicular and membrane trafficking and the innate immune response (Davis and Gack, 2015; Lowrey et al., 2017; Motiam et al., 2020). The PPPDE domain is predicted to homodimerize in which the groove between the dimers constitutes the active site (Suh et al., 2012) and this feature could drive dimerization of the C-termini of VP2 homologs at the surface of the virions (see also below).

### VP2 (A-Spike) as Cell Attachment and Membrane Penetration Protein?

Since VP2 is associated with the turrets, it could have a similar function as the spike protein σ1 of orthoreoviruses which is involved in cell attachment (discussed in section 5). However, VP2 (140 kDa) is much larger than σ1 (50 kDa) and no layer of trimers is found in CPV core particles (Figure 1 and Table 2), which suggests that VP2 may also play a role in membrane penetration. Analogous to σ1, VP2 proteins may be anchored with their N-termini on the virions by association with the turrets, form multimers and extend from the surface to form lollypop structures (see discussion in section 5 on orthoreoviruses). Besides the presence of dimerizing PPPDE domains at the C-termini (see above), coiled-coil regions are predicted at the N-termini, at 1/3rd of protein length and especially at 2/3rd of protein length, that could drive trimerization or tetramerization of A-spike/VP2 proteins and may form filaments (Hartmann, 2017). Other relevant motifs for functioning as membrane penetration proteins, such as furin- or cathepsin-cleavage sites and cell penetrating peptide sequences can also be identified in the primary sequences (Figure 3 and Supplementary Figure 1). Of note, integrin-interacting motifs can be identified in multiple copies of which RGD tripeptides at the exposed C-termini may have special relevance. RGD motifs are found in cell attachment proteins such as fibronectin and typically interact with members of the integrin family of cell adhesion molecules (D’Souza et al., 1991).

### Cypovirus Infection of the Midgut in Lepidoptera

Primary infections by cypovirus virions of columnar epithelial cells of the midgut occur after dissolution of the polyhedra in the alkaline environment of the midgut. In silkworm larvae, cypovirus virions are capable to penetrate directly the cellular membrane of the microvilli in columnar epithelial cells (Tan et al., 2003). Formation of virogenic stroma and production of polyhedra were observed in the columnar cells with characteristic brush border but not in other cell types of the midgut such as goblet cells or associated muscle cells (Tan et al., 2003). While polyhedra can be found in hemolymph and viral RNA can be detected in internal tissues (Kolliopoulou et al., 2015), no other cell types that sustain productive infection of CPV have been identified in the silkworm.

Cytoplasmic polyhedrosis viruses infection of the midgut triggers a strong RNAi response (Zografidis et al., 2015) as well as a complex transcriptional response which includes potential pro-viral and anti-viral genes that remain to be validated by functional assays (review by Swevers et al., 2020). In the midgut, apoptosis and sloughing off of infected cells are considered major pathways of antiviral defense (Gao et al., 2012).

### *In vitro* Models for Cypovirus Infection

Silkworm ovary-derived BmN or Bm5 cells (Wang et al., 2019) have been explored for *B. mori* CPV (BmCPV) production. When dsRNA genomes of BmCPV, isolated from polyhedra from the midgut of larvae, were transfected into Bm5 cells, no pathogenic effects or production of CPV virions could be observed (Swevers et al., 2014). Similarly, transfection of CPV virions isolated from polyhedra did not result in productive infection in the same cell type (Zhao et al., 2019).

In an attempt to develop a reverse genetics system for BmCPV, a mixture of *in vitro* transcribed RNAs corresponding to the different segments of the BmCPV genome was transfected into BmN cells (Guo et al., 2018). While viral gene expression was observed in BmN cells, feeding of cellular extracts of BmN cells transfected with viral RNAs to silkmoth larvae also resulted in the production of viral particles and polyhedra in the midgut (Guo et al., 2018). These experiments indicate that BmN cells may support assembly of virions and polyhedra to a certain extent while amplification of potential recombinant cypoviruses obtained *in vitro* can be achieved in midgut tissue of larvae.

A similar combination of cell line transfection with amplification in larvae was also used to construct a reverse genetics system for *Dendrolimus punctatus* cypovirus (DpCPV; Zhang et al., 2019). In this case, recombinant baculoviruses were employed to express T7 RNA polymerase in lepidopteran Sf9 cells which was used to drive expression of viral RNAs from co-transfected plasmids with T7 polymerase expression cassettes. A deletion of the *vp80* gene was engineered in the baculovirus genome to minimize the effects of baculovirus virion occlusion. CPV polyhedra produced by this expression system were subsequently employed to infect *Spodoptera exigua* larvae by feeding to amplify the embedded recombinant virions that were produced in the cell line (Zhang et al., 2019).

Silkworm BmN cells have also been used to investigate the uptake and intracellular trafficking of BmCPV virions. Cell attachment of BmCPV virions (as released from polyhedra) requires cholesterol and interaction with gangliosides (Zhu et al., 2018) while uptake of BmCPV was carried out by clathrin-mediated endocytosis (Chen et al., 2018). Furthermore, interactions with β-integrins and the scaffolding protein RACK1 were observed (Zhang et al., 2017) and Src64B-like tyrosine kinase also could promote cell entry (Zhang et al., 2018). These results indicate that the uptake mechanism of BmCPV in silkworm cell lines is similar to the uptake of orthoreoviruses in mammalian cell lines (see section 5 on orthoreoviruses), although the molecular details of the interactions in the case of BmCPV need to be clarified.

## Molecular Mechanism of Cell Entry by Birnaviruses

Birnaviruses are dsRNA viruses that infect a variety of animal species, including vertebrates and insects. Birnaviruses that infect insects, such as *Drosophila* X virus (DXV), belong to the *Entomobirnavirus* genus; however, most research has been carried out with Infectious bursal disease virus (IBDV; genus *Avibirnavirus*) that infects birds (Delmas et al., 2019).

The birnavirus genome consists of 2 segments, of which one encodes the viral RNA-dependent RNA polymerase (VP1; 113 kDa in *Drosophila* X virus) and the other the polyprotein pVP2-VP3-VP4. VP4 (28 kDa) is a protease that excises itself and releases pVP2 and VP3. VP3 (29 kDa) forms ribonucleoprotein complexes with dsRNA in the virions and also functions to recruit VP1 to the virion during assembly. pVP2 (48 kDa) is the precursor of the capsid protein which undergoes additional proteolytic processing to mature VP2 protein and smaller peptides that remain associated with the virion (Coulibaly et al., 2005; Valli et al., 2012).

The crystal structure of the IBDV virion reveals a 65–70 nm icosahedral particle with triangulation *T* = 13 that is formed by 260 VP2 trimers (Coulibaly et al., 2005). However, the birnavirus coat protein VP2 has a “hybrid” structure and consists of domains that are found in both (+)-ssRNA and dsRNA viruses. Central in VP2 is the shell (S) domain that has both N- and C-terminal extensions that together form the base (B) domain; together the B and S domains are similar to the capsid proteins of noda- and tetraviruses (which also have bisegmented genomes but of (+)-ssRNA). The projection (P) domain, on the other hand, is inserted in a loop within the S domain and is similar to domains found in μ1, VP6 and VP7, which form the *T* = 13 layer of trimers in orthoreoviruses, rotaviruses and BTV, respectively (Coulibaly et al., 2005). The P domain projects 40 Ǻ from the surface and it can be speculated that VP2 trimers are involved in membrane penetration through their P domains, similar to μ1 trimers in orthoreoviruses and VP7 trimers in ISVPs of BTV (Coulibaly et al., 2005). These considerations point to a conserved role in membrane penetration for the capsid proteins that form the *T* = 13 icosahedral layer of trimers in reoviruses and birnaviruses.

Infectious bursal disease virus causes serious disease in chickens by infecting and destroying developing B lymphocytes in the bursa of Fabricius, the central immune organ for B cell development in birds (Sharma et al., 2000). Several characteristics of cell entry by IBDV have been elucidated (Qin and Zheng, 2017). Cellular factors for virus attachment include Hsp90 located on the cell surface (Lin et al., 2007), the membrane protein Annexin II (Ren et al., 2015), the integrin α4/β1 (potentially through an integrin-interacting motif in VP2; Delgui et al., 2009) and surface immunoglobulin M (Luo et al., 2010). Cellular internalization of IBDV occurs by endocytosis that is clathrin-independent but requires c-Src tyrosine kinase and the actin cytoskeleton as is observed during macropinocytosis (Yip et al., 2012; Gimenez et al., 2015; Ye et al., 2017). IBDV infection is dependent on entry in the early endosomal compartment (characterized by Rab5 small GTPase) (Gimenez et al., 2015) and requires calcium loss and acidification for membrane penetration (Yip et al., 2012). The low calcium concentrations in the endosomal compartment trigger the release of small peptides (pep46 that remains associated with the virion after C-terminal processing of pVP2) from the virions that deform the membrane and lead to the formation of pores (Galloux et al., 2007). The process of endosome permeabilization therefore occurs with a similar mechanism as for the (+)-ssRNA viruses of the families *Nodaviridae* and *Tetraviridae* (Bong et al., 1999).

Bursal disease virus is known to cause B cell apoptosis and suppression of the innate immune response (Qin and Zheng, 2017). Regarding infections of insects with species from the *Entomobirnavirus* genus, DXV triggers the antiviral RNAi response and VP3, a dsRNA-binding protein associated with the birnavirus genome, can function as a suppressor of RNAi (Valli et al., 2012; Van Cleef et al., 2014).

## Implications for the Development of Viral-Like Particles as Carriers and Delivery Vehicles of dsRNA

From the viewpoint of development of vehicles for delivery of dsRNA molecules, reoviruses have several interesting features. Despite not being enveloped by a membrane, reoviruses have acquired properties for efficient transmission of large protein-nucleic acid complexes across the plasma membrane. In addition, they possess large cavities for storage of long dsRNA molecules which are protected by capsid shells that are adapted to resist environmental assaults.

The structural features of dsRNA viruses could therefore be harnessed for the construction of VLPs that function as carriers of dsRNA and that concomitantly contain features for permeabilization of cellular membranes and delivery of cargo into the cytoplasm. To produce VLPs, the baculovirus expression vector system (BEVS) can be considered as a very potent platform because of its capacity for production of large protein complexes (Gopal and Schneemann, 2018; Zhao et al., 2018; Gupta et al., 2019). Since reoviruses and birnaviruses are not enveloped, VLPs may become spontaneously assembled following expression of the constituent capsid proteins (Hagiwara and Naitow, 2003; Kolliopoulou et al., 2017; Zhao et al., 2018). Moreover, since dsRNA viruses that infect insect cells require a much less elaborate composition than is described for infection of mammalian cells, the comparative approach that was followed in this review allows one to make recommendations for the construction of VLPs that combine efficiency of cell entry with relative simplicity of design.

### A Less Elaborate Mechanism for Cellular Entry in Insects

Reovirus virions that infect mammalian cells contain outer capsid layers (Figures 1, 2) that typically carry out a multi-step process to achieve cellular entry: cell attachment, cellular internalization, endosomal trafficking and membrane perforation. The last process is triggered by the insertion of hydrophobic patches in the membrane that result in the formation of pores for entry of the cores (with dsRNA molecules) while the outer capsid layers become dispatched. The molecular details are best understood for rotaviruses but are also worked out well for orthoreoviruses and infection of mammalian cells by BTV (see sections 5, 6 and 7). An overview of the molecular mechanisms of cell entry by the dsRNA viruses that are discussed is presented in Table 1.

On the other hand, evidence has also appeared that the entry of reoviruses in insect cells may be more easily accomplished since virions that infect insect cells have a more simple structure and lack most of the elaborate outer capsid layers that characterize mammalian reoviruses. A clear comparison provides the arbo-reovirus BTV (Figure 2) that is capable to productively infect both mammalian and insect cells (section 7). ISVPs of BTV that are generated following protease treatment of virions are much more infectious in insect than in mammalian cells while core particles can only infect insect cells. Also the plant virus RDV (*Phytoreovirus*) lacks the outer capsid found in BTV (Figure 2), yet can infect efficiently vector cell monolayers (section 8). For both BTV core particles and RDV particles, membrane transition is thought to be mediated by trimer proteins that form a *T* = 13/icosahedral layer (VP7 and P8, respectively). Indeed, the μ1 protein of orthoreoviruses, which is considered homologous to VP7 and P8, has been identified as a penetration protein for orthoreoviruses infections in mammalian cells for which several molecular details have been elucidated (section 5).

Although in RRSV (*Oryzavirus*) the *T* = 13/icosahedral layer of P9 trimers is much reduced (Figure 1), P9, a putative homolog of μ1/VP7/P8, was nevertheless found to be important for infection (section 8). Finally, the capsid proteins of birnaviruses contain a projection domain that is homologous to the trimers of reoviruses for which it is speculated to have a role in membrane penetration (section 10). The available data therefore indicate that the trimers constituting the *T* = 13/icosahedral layer have a role in membrane penetration that is conserved among both non-turreted and turreted reoviruses as well as birnaviruses.

Cypoviruses, on the other hand, are single-layered and lack the *T* = 13/icosahedral layer of trimers (Figure 1). Instead, they possess A-spikes (assembled by VP2) for which it is speculated that they play a role in cell entry (section 9). Large proteins that may function as spikes for cell entry are also found in the turreted plant reoviruses (*Fijivirus* and *Oryzavirus* genera) and mosquito-specific dinovernaviruses (sections 8 and 9). *In silico* analysis of the A-spike of Cypovirus 1 (also known as BmCPV) reveals disorganized regions that are predicted to form coiled-coil structures, a hydrophobic sequence that may function in membrane penetration and potential furin- and cathepsin proteolytic sites (Supplementary Figure 1), which are all features characteristic for forming filaments that could be processed to expose hydrophobic regions. Integrin-interactive motifs that are found at the exposed C-termini (Supplementary Figure 1) may be involved in the initiation of cellular internalization as observed for mammalian reoviruses (sections 5, 6 and 7). More intriguing is the presence of a PPPDE domain that is predicted to have de-ubiquitinating and de-sumoylating activity (section 9). Peptidase activity of this domain may be important for activation of A-spikes for cell entry. Alternatively, it may play a role during virion assembly or as an antiviral defense mechanism. Since the catalytic residues are intact, as a first step the substrate specificity of this domain could be determined to reveal cues about a possible function.

### Design and Composition of “dsRNA-VLPs”

For encapsulation of dsRNA molecules, VLPs will rely on the conserved shell protein (e.g., VP1/CSP of CPV, P3 of RRSV and RDV, VP3 of BTV; Figures 1, 2 and Tables 2, 3) that spontaneously can assemble into icosahedrical particles that naturally encapsulate long dsRNA molecules in dsRNA viruses. Next, such “subcore” or “core” particles need to be endowed with the machinery for penetration of cellular membranes. As already pointed out above, two strategies can be applied: (1) the decoration with a *T* = 13/icosahedral layer of trimer proteins that have the property of membrane destabilization (e.g., P8 of RDV, P9 of RRSV, VP7 of BTV; Figures 1, 2 and Tables 2, 3); or (2) the attachment of spike proteins that can be proteolytically processed to expose hydrophobic regions for membrane penetration (e.g., A-spike/VP2 of CPV, A-spike/P1 of RRSV and P2 of RDV; Figures 1, 2 and Tables 2, 3). The presence of the *T* = 13/icosahedral layer of trimer proteins should be sufficient for efficient uptake in cell lines or after injection in the hemolymph while spike proteins may be necessary for uptake after feeding in the harsh environment of the insect gut (Mertens et al., 1996; Nakagawa et al., 2003). To simplify construction, relevant parts of the spike protein could be rationally inserted in the capsid shell protein such that both carrier and penetration functions are incorporated in a single building block. While this will require extensive engineering, this strategy of simplification has been adopted successfully by birnaviruses (Coulibaly et al., 2005; see section 10).

The capsid protein composition of VLPs must be tailored for their targeting to particular insect groups. For instance, VLPs that target lepidopteran insects should be based on CPV of which the capsid shell protein has specific features for increased stability (Ren et al., 2021); in addition the versatility of the BEVS can be exploited to incorporate VLPs in polyhedra for additional protection in the environment.

An additional requirement is the recruitment of dsRNA cargo into VLPs. DsRNA viruses accomplish the encapsulation of their dsRNA genome segments following the co-assembly of RNA-dependent RNA polymerases that synthesize full-length dsRNA from each ssRNA segment template within the viral subcores or cores (Roy, 2017). However, this strategy is not feasible for VLPs that require incorporation of genetically inert dsRNA molecules. Another possibility would be to express viral capsid proteins in the presence of high concentrations of dsRNA for their passive incorporation during VLP assembly. Nevertheless, while the BEVS is very suitable to drive high levels of protein expression, its capacity for production of long dsRNA molecules seems limited (L.S., A.K., and D.K., unpublished results). More research is required to design strategies for encapsulation of linear dsRNA molecules in VLPs, either by co-assembly or by disassembly-assembly protocols in the presence of high amounts of dsRNA.

### Availability of *in vitro* Models for Functional Testing

A major impediment for the study of cypoviruses (as visualized to function as carriers for dsRNA molecules into lepidopteran insects) is the lack of an efficient reverse genetics system. Two reverse genetics systems have been proposed based on common lepidopteran cell lines but the protocol still requires amplification of potential recombinant virions in the midgut epithelium of larvae (section 9). Infection of the midgut epithelium by CPV presumably occurs at conditions that are difficult to replicate *in vitro* (notably the high pH and the abundant presence of digestive enzymes) and more research efforts should focus on the development of *in vitro* culture models that morphologically and physiologically more closely mimic the insect midgut, for instance by the adaptation of 3D culture conditions and the expansion of organoid technologies to insect cell culture (Swevers et al., 2021). The absence of suitable *in vitro* models for the midgut of lepidopteran insects of course also will affect the reliable testing of “dsRNA-VLPs” that are based on CPV. Mounting of the midgut epithelium in a perfusion chamber has also been described for the study of the barrier function of the insect gut (Cermenati et al., 2011). For now, common lepidopteran cell lines are the most easily accessible *in vitro* system to investigate the uptake mechanism of “passive” (i.e., incapable of robust amplification) virions released from polyhedra (section 9) or CPV-based VLPs that are produced by the BEVS.

While plant reoviruses can be propagated *in vitro* using vector cell monolayers (section 8), reverse genetics systems have not been reported. Interestingly, reoviruses of the *Dinovernavirus* genus can replicate efficiently in mosquito cell lines (*Aedes pseudoscutellaris* reovirus and Fako virus; Attoui et al., 2005; Auguste et al., 2015). Similar to cypoviruses, dinovernaviruses have single-shelled virions with clamps and turrets but lack the *T* = 13/icosahedral layer of trimers (Auguste et al., 2015). Dinovernaviruses have only 9 dsRNA genome fragments and do not form polyhedra. On the other hand, they encode A-spike proteins that are similar to those of cypoviruses (Figure 3 and Supplementary Figure 1). However, A-spikes are not detected in purified virions of Fako virus (Auguste et al., 2015), that could reflect their genuine absence in virions or perhaps loss from the virion surface during the purification procedure. Because an easily accessible *in vitro* infection model is available, the mechanism of infection by dinovernaviruses could be elucidated more easily than for cypoviruses which could also provide insights into the possible role of the A-spikes. Moreover, transfectable cell lines that sustain reproductive infection can be used to develop efficient reverse genetics systems for both plant reoviruses and dinovernaviruses.

### Engineering the Stability of the Core Particle in the Cytoplasm

As a final note, it is clear that the deposition of a large dsRNA-protein complex into the cytoplasm by itself is not sufficient to elicit RNAi. Efficient disassembly of the protein shell and release of free dsRNA are necessary. It has also become apparent that core particles of the Fako virus have a more simple structure and lack several of the stability elements found in cypoviruses (Auguste et al., 2015). Thus, systematic testing could find a balance between protection, penetration and disassembly to design particles that combine penetration potency with efficacy of release of dsRNA.

## Conclusion

Our analysis indicates that insect cells can be infected by reovirus virions with a much more simple structure than is the case for mammalian cells. The question remains what the necessary requirements are for efficient uptake and transport in the cytoplasm and which role is played by the *T* = 13/icosahedrical layer of trimers or the A-spikes. The elucidation of the mechanism requires the availability of suitable reverse genetics systems or biotechnological platforms for the production of VLPs. Testing for internalization and membrane penetration can occur in available cell lines although preferably more suitable *in vitro* midgut models need to be developed. Ultimately, feeding experiments need to be performed to assess the applicability for pest control management.

## Author Contributions

LS and JS conceived the idea and designed the study. LS wrote the first draft of the manuscript. DK, AK, FR, MF, and JS critically read the manuscript and made improvements in the text and the figures. All authors read and approved the final version of the manuscript.

## Conflict of Interest

The authors declare that the research was conducted in the absence of any commercial or financial relationships that could be construed as a potential conflict of interest.

## Publisher’s Note

All claims expressed in this article are solely those of the authors and do not necessarily represent those of their affiliated organizations, or those of the publisher, the editors and the reviewers. Any product that may be evaluated in this article, or claim that may be made by its manufacturer, is not guaranteed or endorsed by the publisher.

## Supplementary Material

Supplementary Figure 1 Sequence alignment of homologs of VP2/A-spike of Cypovirus (Accession number: ALL27239) in other reovirus species that infect insects. To search for VP2 of *BmCPV 1* or *Cypovirus 1* (Accession number: ALL27239) homolog proteins in other insect species a protein–protein BLAST search (BLASTp) was performed (https://blast.ncbi.nlm.nih.gov/). The BLAST searches ran against non-redundant protein sequences using the Blosum 62 matrix (Henikoff and Henikoff, 1992). Only the best hits were used with the lowest *E*-values (one for each organism). Amino acid sequence alignments were performed with the ALIGNW program, a component of the Molecular Evolutionary Genetics Analysis 5 software (Tamura et al., 2011) using the Blosum62 scoring matrix (Henikoff and Henikoff, 1992). Prediction of coiled-coil regions was performed with the Wagga Wagga online tool (Simm et al., 2015). Deduced protein domains were found using the PROSITE online tool (https://prosite.expasy.org/). CPP, cell-penetrating peptide. Three-letter motifs correspond to integrin-interactive motifs.
