# Supplementary material for: Mechanisms of Cell Entry by dsRNA Viruses: Insights for Efficient Delivery of dsRNA and Tools for Improved RNAi-Based Pest Control
Source: Front Physiol. 2021 Nov 11;12:749387. doi: 10.3389/fphys.2021.749387 (PMC8632066; doi:10.3389/fphys.2021.749387)
Supplement: Supplementary Figure 1 — Sequence alignment of homologs of VP2/A-spike of Cypovirus (Accession number: ALL27239) in other reovirus species that infect insects. To search for VP2 of BmCPV 1 or Cypovirus 1 (Accession number: ALL27239) homolog proteins in other insect species a protein–protein BLAST search (BLASTp) was performed (https://blast.ncbi.nlm.nih.gov/). The BLAST searches ran against non-redundant protein sequences using the Blosum 62 matrix (Henikoff and Henikoff, 1992). Only the best hits were used with the lowest E-values (one for each organism). Amino acid sequence alignments were performed with the ALIGNW program, a component of the Molecular Evolutionary Genetics Analysis 5 software (Tamura et al., 2011) using the Blosum62 scoring matrix (Henikoff and Henikoff, 1992). Prediction of coiled-coil regions was performed with the Wagga Wagga online tool (Simm et al., 2015). Deduced protein domains were found using the PROSITE online tool (https://prosite.expasy.org/). CPP, cell-penetrating peptide. Three-letter motifs correspond to integrin-interactive motifs. [file Presentation_1.PPTX]

## Slide 1
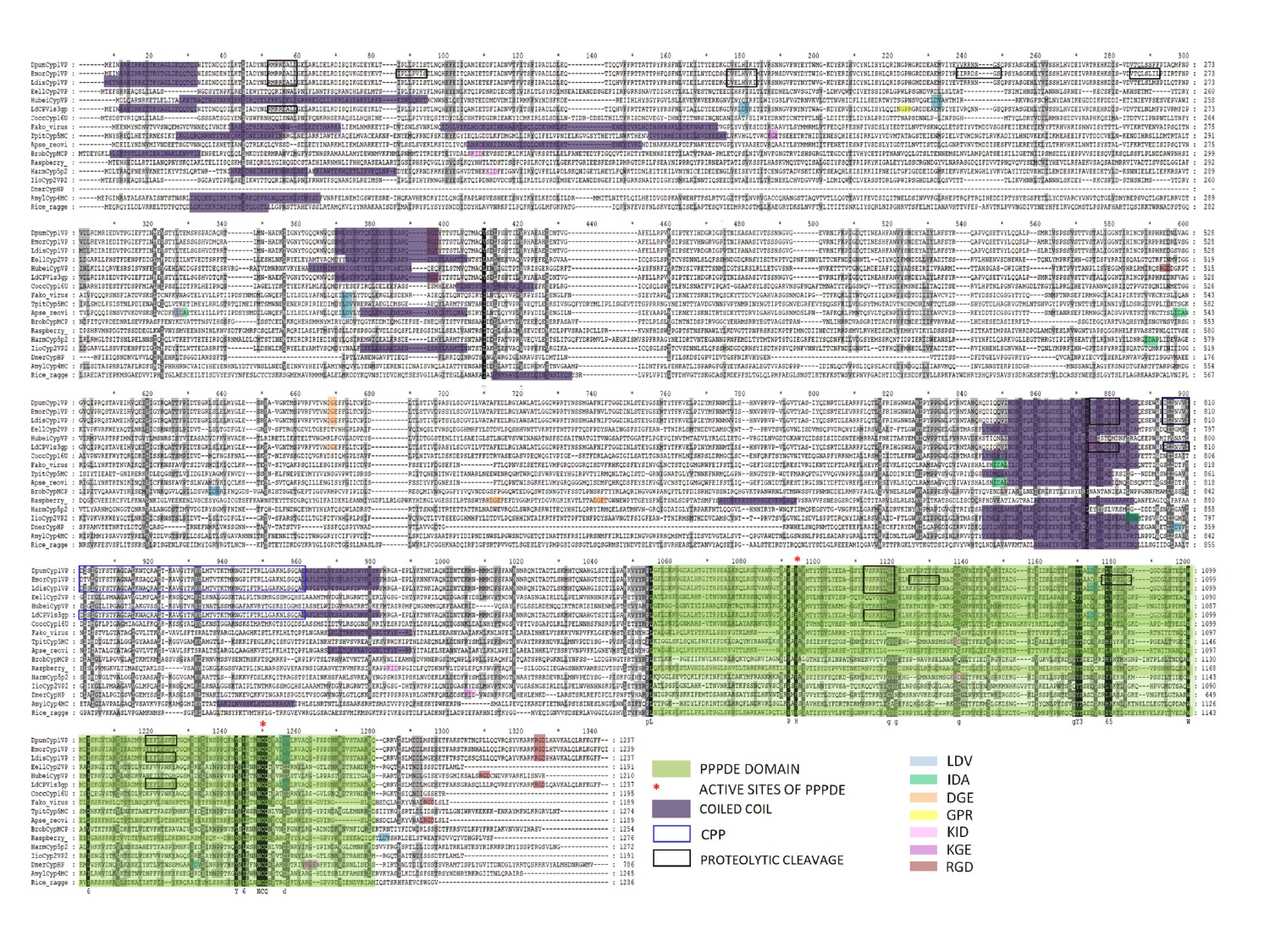

## Slide 2
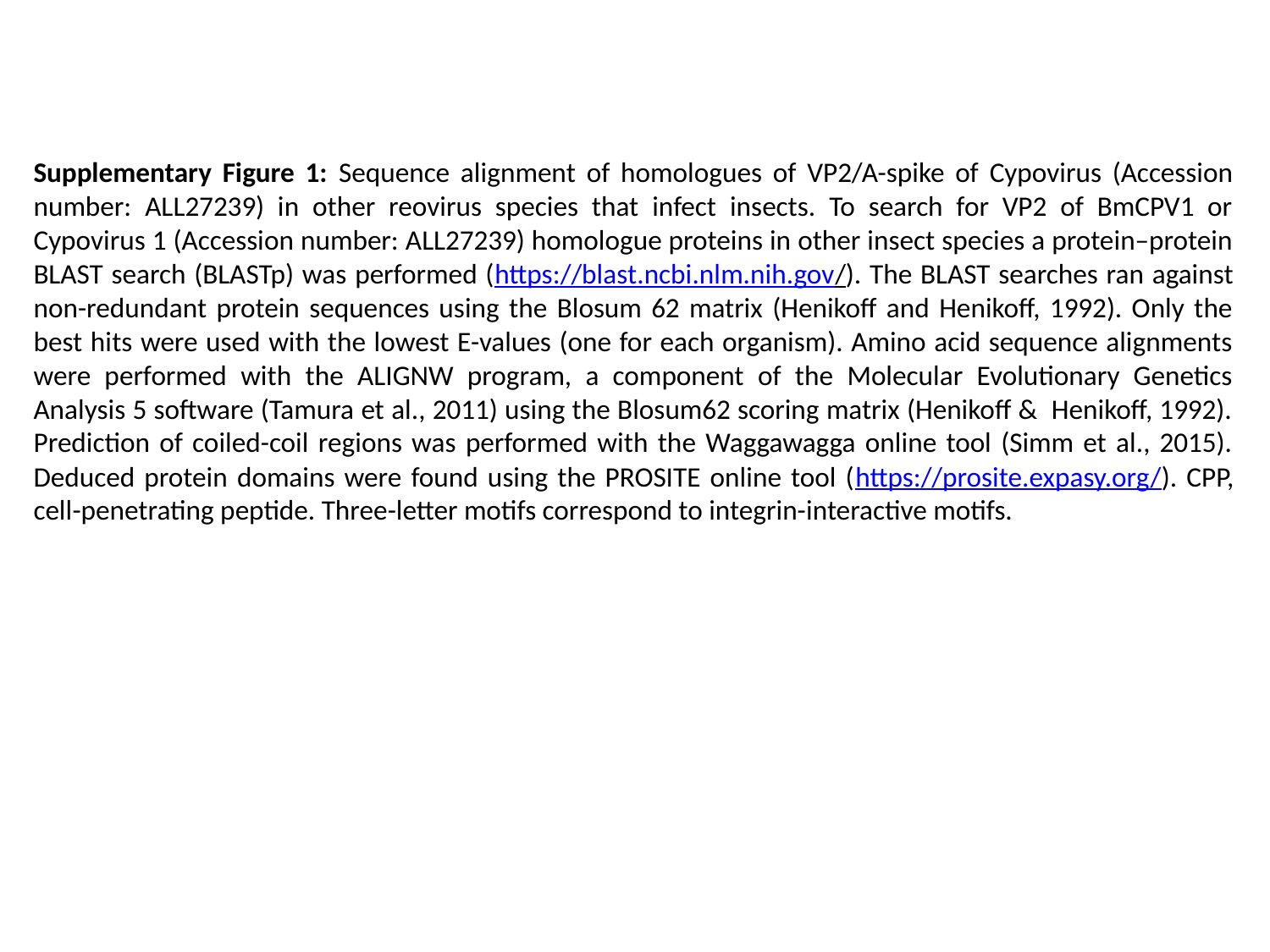

Supplementary Figure 1: Sequence alignment of homologues of VP2/A-spike of Cypovirus (Accession number: ALL27239) in other reovirus species that infect insects. To search for VP2 of BmCPV1 or Cypovirus 1 (Accession number: ALL27239) homologue proteins in other insect species a protein–protein BLAST search (BLASTp) was performed (https://blast.ncbi.nlm.nih.gov/). The BLAST searches ran against non-redundant protein sequences using the Blosum 62 matrix (Henikoff and Henikoff, 1992). Only the best hits were used with the lowest E-values (one for each organism). Amino acid sequence alignments were performed with the ALIGNW program, a component of the Molecular Evolutionary Genetics Analysis 5 software (Tamura et al., 2011) using the Blosum62 scoring matrix (Henikoff & Henikoff, 1992). Prediction of coiled-coil regions was performed with the Waggawagga online tool (Simm et al., 2015). Deduced protein domains were found using the PROSITE online tool (https://prosite.expasy.org/). CPP, cell-penetrating peptide. Three-letter motifs correspond to integrin-interactive motifs.
